# Modulating ion–dipole interactions in nonflammable phosphonate-based electrolyte for safe and stable sodium-ion pouch cells

**DOI:** 10.1093/nsr/nwae466

**Published:** 2024-12-23

**Authors:** Zhuo Yang, Yingying Dai, Zheng-Kun Xie, Shao-Bo Li, Yao-Jie Lei, Jian Chen, Xunzhu Zhou, Zhi-Qiang Hao, Xin Tan, Lin Li, Wei-Hong Lai, Li Li, Wei-Hua Chen, Shu-Lei Chou

**Affiliations:** Institute for Carbon Neutralization Technology, College of Chemistry and Materials Engineering, Wenzhou University, Wenzhou 325035, China; Laboratory of Advanced Materials, Shanghai Key Lab of Molecular Catalysis and Innovative Materials, Fudan University, Shanghai 200438, China; Institute for Superconducting and Electronic Materials, University of Wollongong, Wollongong 2500, Australia; Institute for Carbon Neutralization Technology, College of Chemistry and Materials Engineering, Wenzhou University, Wenzhou 325035, China; College of Chemistry, Zhengzhou University, Zhengzhou 450001, China; School of Materials Science and Engineering, South China University of Technology, Guangzhou 510640, China; Institute for Superconducting and Electronic Materials, University of Wollongong, Wollongong 2500, Australia; Wenzhou Key Laboratory of Sodium-Ion Batteries, Wenzhou University Technology Innovation Institute for Carbon Neutralization, Wenzhou 325035, China; Institute for Carbon Neutralization Technology, College of Chemistry and Materials Engineering, Wenzhou University, Wenzhou 325035, China; Wenzhou Key Laboratory of Sodium-Ion Batteries, Wenzhou University Technology Innovation Institute for Carbon Neutralization, Wenzhou 325035, China; Institute for Carbon Neutralization Technology, College of Chemistry and Materials Engineering, Wenzhou University, Wenzhou 325035, China; Wenzhou Key Laboratory of Sodium-Ion Batteries, Wenzhou University Technology Innovation Institute for Carbon Neutralization, Wenzhou 325035, China; Institute for Carbon Neutralization Technology, College of Chemistry and Materials Engineering, Wenzhou University, Wenzhou 325035, China; Wenzhou Key Laboratory of Sodium-Ion Batteries, Wenzhou University Technology Innovation Institute for Carbon Neutralization, Wenzhou 325035, China; Institute for Carbon Neutralization Technology, College of Chemistry and Materials Engineering, Wenzhou University, Wenzhou 325035, China; Wenzhou Key Laboratory of Sodium-Ion Batteries, Wenzhou University Technology Innovation Institute for Carbon Neutralization, Wenzhou 325035, China; Laboratory of Advanced Materials, Shanghai Key Lab of Molecular Catalysis and Innovative Materials, Fudan University, Shanghai 200438, China; School of Environmental and Chemical Engineering, Shanghai University, Shanghai 200444, China; College of Chemistry, Zhengzhou University, Zhengzhou 450001, China; Institute for Carbon Neutralization Technology, College of Chemistry and Materials Engineering, Wenzhou University, Wenzhou 325035, China; Wenzhou Key Laboratory of Sodium-Ion Batteries, Wenzhou University Technology Innovation Institute for Carbon Neutralization, Wenzhou 325035, China

**Keywords:** sodium-ion battery, solvation chemistry, nonflammable electrolyte, capacity retention, safety

## Abstract

Phosphonate-based electrolytes with the merits of low cost and intrinsic nonflammability are promising candidates to realize the safe operation of sodium-ion batteries. However, they generally suffer from poor interfacial chemistry because of the solvent-dominated solvation structure induced by the strong ion–dipole interactions between cations and phosphonate molecules. Herein, we report an electrolyte design strategy that selectively improves the competitive coordination of low-solvating–power molecules, achieving stable interfacial chemistry with a non-flammable, low-cost and fluorine-free electrolyte. By improving the ion–ion interaction between cation and anion, weakly coordinated molecules can enter the Na^+^ solvation shell, thereby promoting more adjustable and advantageous interfacial chemistry. As a result, the fluorine-free Prussian blue||hard carbon pouch cell, with a high cathode mass loading of ∼20 mg cm^−2^, reaches a high capacity retention with an energy density of over 221.7 Wh kg^−1^ based on electrode mass and 115.1 Wh kg^−1^ based on battery mass.

## Teaser

An electrolyte design based on solvation chemistry regulation has enabled a fluorine-free, high-safety, and long-cycle sodium-ion pouch cell.

## INTRODUCTION

Sodium-ion batteries represent a promising alternative electrochemical energy storage technology to the more expensive, resource-limited lithium-ion batteries, in particular to accommodate the rampant increasing demand for large-scale energy storage systems. Tremendous efforts for electrode materials have been successfully established that can partially fulfill the commercialization of sodium-ion batteries [1–3]. However, most of these battery systems are still based on conventional carbonate-based liquid electrolyte, which remains to have poor thermal stability, high volatility and high flammability, and consequently pose significant safety hazards to the batteries under abusive conditions of mechanical shock, thermal shock, overcharge and short circuit [4–6]. These issues will be amplified as the number and size of sodium-ion batteries developed for large-scale applications increase in the future, which seriously threatens the expansion of this electrochemical energy storage technology.

To mitigate these issues, various non-flammable liquid electrolyte designs have been reported, including phosphonate-based electrolytes, nitrile-based electrolytes, fluorinated electrolytes, ionic liquid electrolytes, etc. Among various reported options, phosphonate solvents represented by trimethyl phosphonate (TMP) and triethyl phosphonate (TEP) remain the preferred choice for designing non-flammable electrolyte due to their numerous benefits including low cost, low toxicity, intrinsic nonflammability, low volatility and wide liquid-phase temperature ranges [7–10]. However, the conventional TMP- and TEP-based electrolyte fail to match the carbon-based electrode, leading to electrolyte depletion, low Coulombic efficiency, irreversible capacity loss and shortened cycle life [11–14]. This arises because of the strong ion–dipole interactions between cations and phosphonate molecules, which result in the formation of TMP- or TEP-dominated solvation structures. As the precursor of electrode-electrolyte interphase (EEI), the solvation structure is closely related to interfacial chemistry [15–17]. This TMP- and TEP-dominated solvation ultimately leads to the development of an unstable EEI due to the continuous decomposition of phosphonate molecules, thereby compromising the electrochemical performance of the system.

To address these concerns, introducing alternative ionic or molecular ligands into the solvation sheath to reduce phosphonate molecules and form an anion-rich or co-solvent-rich solvation structure has been considered as a rational strategy [18,19]. Various advanced electrolyte design strategies, including the use of high-concentration fluorinated salts or high content of co-solvents (such esters or ethers) have attempted to induce a stable anion-derived or co-solvent-derived interfacial chemistry [20–24]. Nevertheless, the anion-derived and co-solvent-derived interfacial chemistry required a heavy use of fluorinated salts and flammable co-solvents, which inevitably leads to escalated production cost and increased risk of electrolyte flammability [25–28]. Given these factors, designing electrolytes with inherently safe physical properties and beneficial interfacial chemistry to ensure a highly reversible and stable electrode reaction is seemingly insurmountable for sodium-ion batteries.

Herein, we report that the ion–dipole interactions of different electrolyte components can be specifically enhanced through the modulation of ion–ion interactions, enabling tunable and advantageous interfacial chemistry. A series of cyclic and linear carbonate molecules were separately introduced as additives (the additive amount is 5 wt%) in NaClO_4_-TEP solution to formulate a fluorine-free non-flammable electrolyte for high-safety, high–energy-density and long–cycle-life sodium-ion batteries. We found that the enhancement of Na^+^–ClO_4_^−^ interactions can tailor the different ion–dipole interactions (Na^+^-phosphonate molecules and Na^+^-carbonate molecules), which modifies the coordination number of carbonate molecules in the Na^+^ solvation structure and facilitates the formation of a robust mosaic-type EEI derived from carbonate compounds. As a result, the fluorine-free rhombohedral Prussian blue (RPB)||commercial hard carbon (HC) pouch cell with designed 2 M NaClO_4_-TEP/vinylene carbonate (VC) electrolyte, enabling high capacity retention of 96% after 50 cycles at 50 mA g^−1^, and high energy density of over 221.7 Wh kg^−1^ based on cathode and anode mass and 115.1 Wh kg^−1^ based on total battery mass. This work highlights the regulation of solvation structure toward developing practical, safe and stable fluorine-free sodium-ion batteries with superior electrochemical performance.

## RESULTS AND DISCUSSION

### Design principle of electrolyte

**Figure 1.**
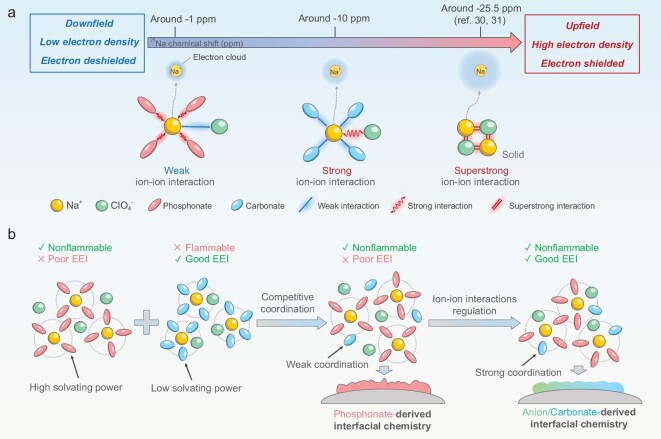
Electrolyte design principles. (a) Schematic illustration of the relationship between ^23^Na chemical shift and Na^+^ coordination environment. (b) Schematic illustration of the transformation of solvation structure and interfacial chemistry.

Nuclear magnetic resonance (NMR) has proven to be one of the most powerful analysis techniques for monitoring the solvation structure of electrolyte due to its ability to provide convincing evidence through the more site-specific probing of atom nuclei. Fig. 1a exhibits the ^23^Na chemical shift in different coordination environments of Na^+^ ions. Notably, the Na^+^ ions in carbonate-based electrolyte exhibit a lower chemical shift (around −10 ppm) compared to that in phosphonate-based electrolyte (around 0 ppm), indicating stronger coordination of the ligands surrounding Na^+^ ions in carbonate-based electrolyte (see Fig. S1 in Supplementary data). This is attributed to the stronger ion–ion interaction between Na^+^ ions and ClO_4_^−^ anions in carbonate solvents compared to phosphonate solvents [29], which cannot effectively separate cations and anions, resulting in more ClO_4_^−^ anions to coordinate with Na^+^ cations. In the most extreme case, ClO_4_^−^ anions directly coordinate with Na^+^ cations to form NaClO_4_ solids, wherein the ^23^Na chemical shift exhibits the minimum value (around −25.5 ppm) [30,31]. Thus, it can be inferred that the solvation structure of carbonate-based electrolyte tends to promote the formation of anion-rich solvation structures more easily than that of phosphonate-based electrolyte [32].

The competitive coordination between solvent and anion ligands in the solvation structure plays a key role in interfacial chemistry. As shown in Fig. 1b, due to the strong solvation ability of phosphonate molecules, the conventional phosphonate-based electrolyte, which is dominated by solvent-separated ion pairs (SSIP) solvation structure, leads to a phosphonate-derived interfacial chemistry. This interfacial chemistry generally causes continuous decomposition of solvent, limiting its practical application. Previous studies demonstrate that introducing benign co-solvents (such esters or ethers) into the solvation sheath to reduce the amount of phosphonate molecules can induce a stable co-solvent derived interfacial chemistry, which significantly suppresses adverse side reactions of phosphonate. However, owing to the high solvating power of phosphonate molecules, the conventional co-solvent derived interfacial chemistry relies on the extensive use of flammable benign co-solvents to competitively coordinate into the primary solvation shell, which inevitably exacerbates the flammability risk associated with the electrolyte. Here, we modulate the ion–ion interaction to enhance the competitive coordination ability of low–solvating-power molecules within a high–solvating-power solvent, thereby effectively tailoring the formation of EEI. Based on this, the carbonate molecules can enter the primary solvation sheath even at low concentrations, forming a carbonate-derived EEI to prevent the subsequent degradation of phosphate molecules. As a result, such carbonate-derived passivation films contain stable and uniform organic-inorganic Na-based compounds, protecting the electrode from further detrimental interfacial side reactions caused by the decomposition of phosphonate molecules, thus enhancing the cyclability of batteries. Herein, a fluorine-free non-flammable electrolyte consisting of 2 M NaClO_4_ in TEP with 5 wt% VC is formulated with a cost of only ∼0.074$ g^−1^, much lower than that of ∼0.314$ g^−1^ for commercial 1 M NaPF_6_ in ethylene carbonate/diethyl carbonate (EC/DEC, with vol% of 1:1) with 5 wt% fluoroethylene carbonate (FEC), according to the data in Fig. S2. The flammability of electrolytes was quantitatively evaluated by self-extinguishing time and a more stringent closed-cup flash point test (see Figs S3, S4 and Videos S1–S3). The 2 M NaClO_4_-TEP/VC electrolyte endows a low self-extinguishing time (SET) of 0 s g^−1^ and a high closed-cup flash point of 141°C, which is distinguished from the commercial 1 M NaPF_6_-EC/DEC/FEC electrolyte with over 20 s g^−1^ and 47°C, respectively. The thermogravimetric curve of 2 M NaClO_4_-TEP/VC electrolyte exhibits a negligible mass loss of ∼3 wt% (∼38% for 1 M NaPF_6_-EC/DEC/FEC electrolyte) at 100°C, indicating its superior thermal stability (see Fig. S5). Furthermore, the measurements of ionic conductivity, wettability and electrochemical stability window were also conducted to assess the properties of 2 M NaClO_4_-TEP/VC electrolyte, which exhibit good practicability and potential for large-scale applications (see Figs S6–S8). These results demonstrate that the electrolyte comprising of 2 M NaClO_4_-TEP/VC electrolyte possesses remarkable thermal stability and nonflammability, along with impressive comprehensive physicochemical characteristics, showcasing the appealing potential for practical application in sodium-ion batteries.

### Analysis of solvation structure

**Figure 2.**
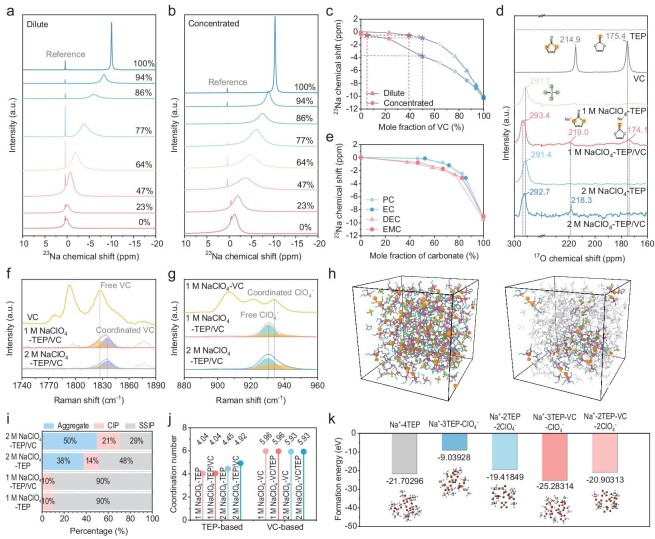
Experimental and theoretical analysis of the solvation structure. (a) The displacement of ^23^Na NMR chemical shifts with the change of VC content in (a) dilute (0.1 M) and (b) concentrated (1 M) NaClO_4_-TEP/VC electrolyte. Mole fraction dependence of the ^23^Na NMR chemical shifts in (c) TEP-VC mixtures and (d) TEP-EC/PC/DEC/EMC mixtures (the concentration of NaClO_4_ is 0.1 M). (e) The ^17^O-NMR spectra of different components and electrolytes. Deconvolution analyses of (f) VC and (g) ClO_4_^−^ Raman spectra signal from different electrolytes. (h) Snapshots of the MD simulation boxes of 2 M NaClO_4_-TEP/VC (left) and corresponding VC-rich solvation structure (right). (i) The proportions of aggregate, CIP, and SSIP solvation structure in each electrolyte. (j) The total coordination number of TEP-based (VC as 5wt% additive) and VC-based (TEP as 5wt% additive) electrolytes. (k) Fomation energy of different solvation structures.

The solvation structure was investigated by spectroscopic characterizations combined with first-principles calculation. As shown in Fig. 2a–c, Na^+^ ions experience a significant upfield shift as the VC mole fraction increases from 0% to 100% in both dilute and concentrated electrolytes, indicating a high electron density around the Na nucleus caused by strong coordination of surrounding ligands. Similar phenomena can also be observed in other carbonate solvents (Fig. 2d and Fig. S9). Given the weaker solvation ability of carbonate than TEP molecules, the obvious displacement is not attributed to the ion–dipole interaction between Na^+^ and carbonate molecules. Instead, the enhanced electron density is due to the ion–ion interaction between Na^+^ and ClO_4_^−^ ions after the introduction of VC. Interestingly, the change in the ^23^Na chemical shift becomes increasingly pronounced with a higher VC content in concentrated electrolytes. For instance, the ^23^Na chemical shift in dilute electrolyte is ∼0.9 ppm at a VC mole fraction of 50%, in contrast, it is ∼3.7 ppm in a concentrated electrolyte (Fig. 2c, blue point). Accordingly, the addition of only 5% mole fraction of VC in concentrated electrolyte can form a comparable coordination strength to that achieved by adding 40% mole fraction of VC to a dilute electrolyte (Fig. 2c, red point). These results indicate that low–solvating-power molecules, such as carbonate, can regulate solvation structures more effectively and induce various interfacial chemistries in the presence of an enhanced ion–ion interaction. This enhancement can also be confirmed in ^17^O-NMR (Fig. 2e and Fig. S10), which showed a noticeable downfield shift of ^17^O signals (ClO_4_^−^ and VC) in TEP/VC-based electrolyte, indicating an enhanced electron density around the O nucleus due to the stronger affinity between Na^+^ and ClO_4_^−^ ions. As shown in Fig. 2f, g and Fig. S11, Raman spectra reveal that the characteristic peaks at 1827.21 and 930.44 cm^−1^ correspond to the C=O stretching vibration of VC molecules and symmetric stretching vibration of ClO_4_^−^, respectively (Fig. 2f, g). The slightly increased peaks of coordinated VC and coordinated ClO_4_^−^ appear at 1835.63 and 934.26 cm^−1^. In 1 M NaClO_4_-TEP/VC electrolytes, the percentages of VC and ClO_4_^−^ coordinated with Na^+^ are 56% and 34%, respectively. However, in the 2 M NaClO_4_-TEP/VC electrolyte, the percentage of ClO_4_^−^ coordinated with Na^+^ increases to 46%, while 72% of VC is involved in the solvation structure. This suggests that the enhanced Na^+^–ClO_4_^−^ interactions also strengthen the interaction of Na^+^–VC pairs.

Classical molecular dynamic (MD) and density functional theory (DFT) calculations further confirmed the experimental evidence. The snapshots of the simulated 2 M NaClO_4_-TEP/VC electrolyte displayed abundant characteristic aggregate and contact ion pairs (CIP), which are predominantly composed of VC-rich solvation structures (Fig. 2h). By contrast, the solvation structure of aggregate and CIP is significantly reduced in 2 M NaClO_4_-TEP compared to 2 M NaClO_4_-TEP/VC (see Figs S12 and S13). Specifically, the solvation structure in the 2 M NaClO_4_-TEP/VC electrolyte exhibits a higher proportion of 50% for aggregate and 21% for CIP, surpassing the 38% aggregate and 14% CIP proportions in the 2 M NaClO_4_-TEP electrolyte. The proportions of free VC/coordinated VC and free ClO_4_^−^/coordinated ClO_4_^−^ reveals the presence of tightly coordinated VC and ClO_4_^−^ ligands surrounding Na^+^ ions in 2 M NaClO_4_-TEP/VC electrolyte (Fig. 2i), which is unattainable in 1 M NaClO_4_-TEP/VC electrolyte and 2 M NaClO_4_-TEP electrolyte (see Fig. S14). To further investigate the effects of the enhanced ion–ion interactions, radial distribution function (RDF) analysis was performed to evaluate the total coordination number of TEP-based and VC-based electrolyte. As shown in Fig. 2j and Figs S15, S16, the total coordination number of Na^+^ in TEP-based electrolyte exhibits a pronounced sensitivity to the change of ion–ion interactions, whereas such an effect is less pronounced in VC-based electrolyte. This may be attributed to the unique high solvating power and large steric hindrance of TEP molecules leading to the unsaturated coordination of Na^+^ ions. As the ion–ion interactions increase, VC molecules with lower solvating power and smaller steric hindrance can enter the Na^+^ primary solvation sheath, ultimately leading to an enhancement in the total coordination number of Na^+^ ions within the TEP-based electrolyte. This can also be confirmed in the comparison of formation energies of different Na^+^ solvation structures. As shown in Fig. 2k, compared to the Na^+^-3TEP-ClO_4_^−^ and Na^+^-2TEP-2ClO_4_^−^ solvation structure in 2 M NaClO_4_-TEP electrolyte, the VC-rich Na^+^-3TEP-VC-ClO_4_^−^ and Na^+^-2TEP-VC-2ClO_4_^−^ solvation structure in 2 M NaClO_4_-TEP/VC electrolyte have a lower formation energy, demonstrating that the VC-rich solvation structure is more prone to formation within the 2 M NaClO_4_-TEP/VC electrolyte. In addition, the narrower highest occupied molecular orbital–lowest unoccupied molecular orbital (HOMO–LUMO) energy gap of the VC-rich solvation structure in 2 M NaClO_4_-TEP/VC electrolyte than that in 2 M NaClO_4_-TEP electrolyte (see Figs S17 and S18), which is more prone to gaining or losing electrons, thus taking precedence over decomposing and forming solid electrolyte interphase (SEI) and cathode electrolyte interphase (CEI). Therefore, the Na^+^ primary solvation sheath with more carbonate molecules through the enhanced ion–ion interactions, which integrated various attractive properties, persuades us to conduct a more comprehensive assessment of its electrochemical performance in complete battery systems.

### Electrochemical performance of sodium-ion full cells

**Figure 3.**
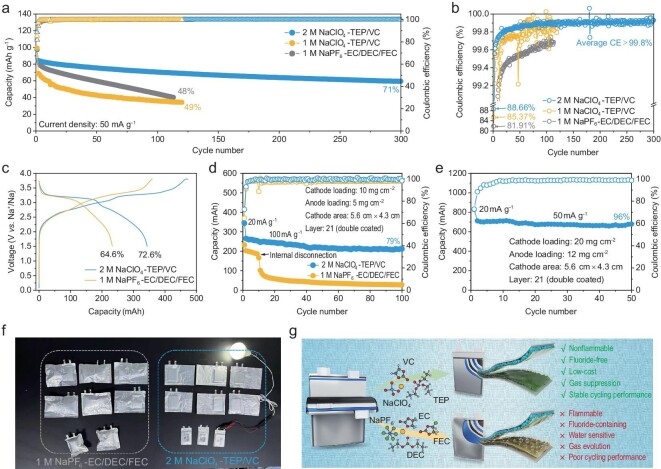
Electrochemical performance of RPB||HC full cells using different electrolytes. (a) Cycling performance of RPB||HC coin cells using different electrolytes cycled at 50 mA g^−1^ after the first activation cycle at 20 mA g^−1^. (b) The comparison for Coulombic efficiency of RPB||HC full cells in different electrolytes. (c) The initial charge-discharge curves of RPB||HC pouch cells (the mass loading of cathode and anode is 10 and 5 mg cm^−2^ for single side, respectively) with 2 M NaClO_4_-TEP/VC and 1 M NaPF_6_-EC/DEC/FEC. (d) Corresponding cycling performance of RPB||HC pouch cells at 100 mA g^−1^ after the first activation cycle at 20 mA g^−1^. (e) Cycling performance of RPB||HC pouch cells with higher mass loading of cathode (20 mg cm^−2^ for single side) and anode (12 mg cm^−2^ for single side) using 2 M NaClO_4_-TEP/VC electrolyte after the first activation cycle at 20 mA g^−1^. (f) Digital photograph of the comparison for gas generation after charge and discharge of RPB||HC pouch cells with 1 M NaPF_6_-EC/DEC/FEC (left) and 2 M NaClO_4_-TEP/VC (right). (g) Comparison of the properties of pouch cells with conventional 1 M NaPF_6_-EC/DEC/FEC electrolyte and 2 M NaClO_4_-TEP/VC electrolyte.

In order to validate the feasibility of 2 M NaClO_4_-TEP/VC electrolyte, sodium-ion full cells were constructed using the RPB cathode and commercial HC anode with a high electrode loading (10 mg cm^−2^ for the cathode and 5 mg cm^−2^ for the anode). The long-term cycling performance of RPB||HC with different electrolytes is compared in Fig. 3a. In the first cycle of activation, the RPB||HC cells with 2 M NaClO_4_-TEP/VC electrolyte exhibit a high initial Coulombic efficiency of 88.8% at 20 mA g^−1^, which are higher than that with 1 M NaPF_6_-EC/DEC/FEC (85.2%) and 1 M NaClO_4_-TEP/VC (85.8%), suggesting the cells with 2 M NaClO_4_-TEP/VC electrolyte possess superior reversibility with less of a side reaction. In the following cycles, there is continuous capacity decay to ∼40 mAh g^−1^ after 110 cycles in both 1 M NaPF_6_-EC/DEC/FEC and 1 M NaClO_4_-TEP/VC electrolyte, with a lower average Coulombic efficiency than that of 2 M NaClO_4_-TEP/VC electrolyte (Fig. 3b). Comparing the charge-discharge curves and electrochemical impedance spectrum of the RPB||HC cells with different electrolytes, the cells in 1 M NaPF_6_-EC/DEC/FEC show a continuous capacity fading throughout the whole cycle without significant increase in resistance, but 1 M NaClO_4_-TEP/VC electrolytes exhibit more capacity loss in the early cycles along with an obvious increase in voltage polarization and resistance (see Figs S19 and S20). The decay mechanism in 1 M NaPF_6_-EC/DEC/FEC may be caused by poor EEI formation, whereas the decay in 1 M NaClO_4_-TEP/VC electrolyte may be attributed to the high-impedance products formed by continuous decomposition of the solvents [33–35], which impede the transport of Na^+^. This can also be demonstrated by the significant difference in reversible capacity and voltage polarization of cathode and anode half cells (see Fig. S21). In contrast, the full cells in 2 M NaClO_4_-TEP/VC electrolyte with low voltage polarization present the best cycling stability, retaining 71% of the original reversible capacity after 300 cycles, and an average Coulombic efficiency of ∼99.8%. This could be attributed to the successful formation of a VC-rich solvation structure with more ClO_4_^−^ and VC occupying the inner sheath in 2 M NaClO_4_-TEP/VC electrolyte, inducing a robust anion-/carbonate-derived passivation film, which effectively avoids the irreversible capacity loss during cycling in RPB||HC cells. The excellent compatibility of 2 M NaClO_4_-TEP/VC electrolyte can also be demonstrated by the rate performance at room temperature and cycling performance at −20°C and 55°C, which indicate fast charge transfer at the EEI with wider temperature endurance (see Figs S22 and S23). Differential capacity (d*Q*/d*V*) analysis and cyclic voltammetry (CV) were carried out for clarifying the charging/discharging process of RPB||HC cells in different electrolytes (see Figs S24 and S25). It can be found that a pair of peaks between 2.8 and 3.4 V, representing the reversible transformation of different Fe-3d states on the C and N sites in the RPB cathode and the sodiation/de-sodiation process of the HC anode [36]. An apparent capacity loss could be observed in both d*Q*/d*V* and CV curves of 1 M NaClO_4_-TEP/VC electrolyte, indicating that the function of VC additives is a failure due to the formation of deteriorative passivation film on the surface of the electrode or the aggressive solvation consumption. These results suggest that the carbonate-rich solvation structure is beneficial for the improvement of electrochemical performance.

To further confirm the practicability of 2 M NaClO_4_-TEP/VC electrolyte, the RPB||HC pouch cells with two different mass loadings and a double-face coating were fabricated. The dimensions, practical weight and components of the pouch cells are shown in Figs S26 and S27. When using a cathode mass loading of 10 mg cm^−2^ and an anode mass loading of 5 mg cm^−2^, the RPB||HC pouch cell with 2 M NaClO_4_-TEP/VC electrolyte exhibited a high discharge capacity of 343.7 mAh and initial Coulombic efficiency of 72.6% at 20 mAh g^−1^, which are higher than that with conventional electrolyte of 1 M NaPF_6_-EC/DEC/FEC (232.6 mAh and 64.6%, respectively, Fig. 3c). This can be explained by the higher irreversible decomposition and lower reversibility of active Na^+^ in 1 M NaPF_6_-EC/DEC/FEC electrolyte than that in 2 M NaClO_4_-TEP/VC electrolyte during the first cycle. During the following cycles, the RPB||HC pouch cells with 1 M NaPF_6_-EC/DEC/FEC electrolyte show a significant capacity fading after 10 cycles due to the poor contact caused by severe gas generation (Fig. 3d) [37,38]. In sharp contrast, the RPB||HC pouch cell with 2 M NaClO_4_-TEP/VC electrolyte exhibits high voltage retention of 99.1% after 30 days and superior capacity retention of 79% (excluding the first cycle of activation) with average Coulombic efficiency >99.5% after 100 cycles (Fig. 3d and Fig. S28), which could be ascribed to the good compatibility of the fluorine-free non-flammable electrolyte with the electrode and the robust EEI layer formation on the electrode surface. Building upon the superior performance achieved with a cathode mass loading of 10 mg cm^−2^ and anode mass loading of 5 mg cm^−2^, we tested the electrochemical performance of 2 M NaClO_4_-TEP/VC electrolyte in the RPB||HC pouch cell with a higher cathode and anode mass loading. As shown in Fig. 3e and Fig. S29, the RPB||HC pouch cell with an ultrahigh cathode mass loading of 20 mg cm^−2^ and anode mass loading of 12 mg cm^−2^ can steadily operate at 50 mA g^−1^ for 50 cycles with high capacity retention of 96% (excluding the first cycle of activation). Although this RPB||HC pouch cell based on the 2 M NaClO_4_-TEP/VC electrolyte is just a handmade prototype without any other structural or technological optimizations, this pouch cell was able to produce an energy density of 221.7 Wh kg^−1^ based on electrode mass and 115.1 Wh kg^−1^ based on total battery mass with high safety performance (Video S4, Supporting Information).

Furthermore, we observed significant gas production in RPB||HC pouch cells when using 1 M NaPF_6_-EC/DEC/FEC electrolyte; however, this cannot be observed in coin cells (Fig. 3f). Even if the gas is released after battery activation, a large amount of gas will still be generated in the subsequent cycle (see Figs S30 and S31), which indicates the successive electrolyte decomposition with severe capacity decay of RPB||HC pouch cells with 1 M NaPF_6_-EC/DEC/FEC electrolyte. In contrast, although previous studies have reported that phosphonate-based electrolyte can produce mixed gas including CO, CO_2_ and H_2_ [38], there is no gas production in the 2 M NaClO_4_-TEP/VC system during the whole of the battery cycle, indicating that the decomposition of TEP is effectively prevented by the robust passivation film. The comparison of the practical considerations concerning the conventional 1 M NaPF_6_-EC/DEC/FEC and 2 M NaClO_4_-TEP/VC electrolyte can be found in Fig. 3g. Compared with conventional carbonate electrolyte, the pouch cell with 2 M NaClO_4_-TEP/VC electrolyte is not only non-flammable, but also has stable cycle performance without gas generation during the whole cycle. These results further verify the practical feasibility of the 2 M NaClO_4_-TEP/VC electrolyte.

The universal verification of this electrolyte design strategy was further confirmed by using other carbonate molecules as additives. As shown in Figs S32–S34, both the RPB||HC coin and pouch cells exhibit a high reversible capacity and Coulombic efficiency when using 2 M NaClO_4_-TEP/EC and 2 M NaClO_4_-TEP/PC electrolyte, and maintain good capacity retention. Similar to VC, this implies that the carbonate-rich solvation structure is effectively achieved by enhanced ion–ion interactions, thereby facilitating a stable anion-/carbonate-derived interfacial chemistry to ensure the long-term stability of battery cycling. It is worth noting that the RPB||HC cells experience rapid capacity decays and low Coulombic efficiency in both 2 M NaClO_4_-TEP/ethyl methyl carbonate (EMC) and 2 M NaClO_4_-TEP/diethyl carbonate (DEC) electrolytes, which is similar to the performance seen in the pure 2 M NaClO_4_-TEP electrolyte with no addition of additives. This phenomenon could be attributed to the relatively inferior passivation ability of linear carbonates in comparison to cyclic carbonates. Despite an anion-/carbonate-derived interfacial chemistry being formed, linear carbonates are unable to adequately passivate the electrode as effectively as cyclic carbonates, ultimately resulting in poor electrochemical performance. Therefore, the results confirmed the synergistic effect between the anion and carbonate additives in improving EEI stability.

### Characterization of the formation of CEI and SEI

**Figure 4.**
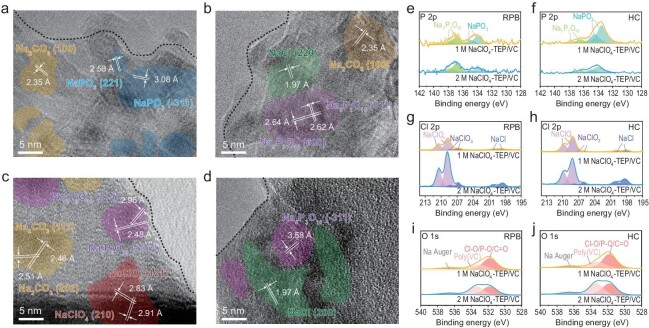
CEI and SEI components and structures of the cycled RPB and HC electrodes. Cryo-TEM images of (a, b) RPB cathodes and (c, d) HC anodes after being cycled in 2 M NaClO_4_-TEP/VC. The P 2p, Cl 2p and O 1s XPS spectra on the (e, g, i) cycled RPB cathodes and (f, h, j) cycled HC anodes.

The reason for the excellent electrochemical performance of 2 M NaClO_4_-TEP/VC electrolyte was further disclosed by investigating the interfacial chemistry caused by the unique VC-rich solvation structure. Therefore, CEI and SEI in the electrolyte were examined using scanning electron microscopy (SEM), cryo-transmission electron microscopy (TEM), X-ray photoelectron spectroscopy (XPS) and time-of-flight secondary ion mass spectroscopy (TOF-SIMS) characterization to investigate the chemical composition and internal structure. The analysis was performed after 50 cycles of RPB||HC full cell with a current density of 50 mA g^−1^. The SEM images of cycled RPB and HC electrodes show that the RPB cathode and HC anode morphology in 2 M NaClO_4_-TEP/VC electrolyte is cleaner and is most similar to the pristine electrode surface (see Fig. S35). In contrast, there are numerous small particles aggregating on the electrode surfaces with 1 M NaClO_4_-TEP/VC and 1 M NaPF_6_-EC/DEC/FEC electrolyte, which may be caused by the unfavorable decomposition of the electrolyte. As shown in Fig. S36, the high-resolution cryo-TEM images of these cycled electrodes further show that a regular and homogeneous EEI with a thickness of 25 and 20 nm formed on the surface of RPB and HC using 2 M NaClO_4_-TEP/VC electrolyte, respectively, which are more uniform and thinner than that formed using 1 M NaClO_4_-TEP/VC and 1 M NaPF_6_-EC/DEC/FEC electrolyte. Fig. 4a–d reveals that the CEI and SEI in 2 M NaClO_4_-TEP/VC electrolyte exhibit a mosaic-type structure with Na_2_CO_3_, NaCl, NaClO_4_, NaPO_3_ and Na_5_P_3_O_10_ nanocrystalline dispersed in an amorphous organic matrix, and these components with high crystallinity can clearly be identified by corresponding fast Fourier transform (FFT) results (see Fig. S37). The formation of NaPO_3_ and Na_5_P_3_O_10_ nanocrystalline can be attributed to the decomposition and polymerization of TEP, while Na_2_CO_3_ stems from the byproduct of VC decomposition. It is worth noting that the amorphous organic matrix in 2 M NaClO_4_-TEP/VC electrolyte observed on electrodes can be attributed to the polymeric-type species (mainly poly(VC)), which results from the decomposition of VC [39]. These polymeric-type species exhibit a binding property that enables them to uniformly coat the electrode surface and bind with other inorganic components. Despite being thicker than the high fluorinated interphase reported previously, the results of electrochemical performance indicate that this inorganic–organic mixed EEI possesses good stability and ion conductivity. In contrast, the CEI and SEI in 1 M NaClO_4_-TEP/VC electrolyte with a TEP-dominated solvation structure showed a monolithic structure comprising bulk inorganic species NaPO_3_ and Na_2_CO_3_ (see Figs S36 and S38). This is attributed to the decomposition of TEP molecules caused by continuous electron attack [40], and resulting in high impedance and consumption of active Na^+^. In 1 M NaPF_6_-EC/DEC/FEC electrolyte, there is a typical dual-layer structure with an amorphous matrix as the outer layer and stiff inorganic compounds such as NaF and Na_2_CO_3_ as the inner layer which has been reported in previous studies (see Figs S36 and S39) [14].

XPS characterization was applied to validate the elemental composition obtained from cryo-TEM results (Fig. 4e–j and Fig. S40). As shown in Fig. 4e–h, the deconvolution of P 2p and Cl 2p spectra revealed several pairs of doublets, representing NaPO_3_ (at around 130.5–135.7 eV), Na_5_P_3_O_10_ (at around 135.5–138.4 eV), NaCl (at around 196.7–201.3 eV), NaClO_3_ (at around 205–209 eV) and NaClO_4_ (at around 206.6–211.5 eV), respectively, which was consistent with the cryo-TEM and FFT results. In 2 M NaClO_4_-TEP/VC electrolyte, the peak intensities of NaPO_3_ in the surface of RPB and HC are lower than that of 1 M NaClO_4_-TEP/VC, but the peak of Cl-based components showed higher intensity, indicating that the decomposition of TEP is effectively suppressed. Moreover, the O 1s spectra of RPB and HC using 2 M NaClO_4_-TEP/VC electrolyte shows that the peak at ∼533.4 eV is higher than that of 1 M NaClO_4_-TEP/VC electrolyte, which is ascribed to the formation of poly(VC) caused by the further polymerization of VC (Fig. 4i, j). The O 1s peak at ∼532.0 eV is most likely from P–O, Cl–O and C=O species generated by incomplete decomposition of TEP and ClO_4_^−^, which is difficult to distinguish from each other due to significant overlap. These results can be also confirmed by the atomic concentration of each electrode, where the RPB and HC in 2 M NaClO_4_-TEP/VC electrolyte exhibit higher content of O and Na but lower content of P as compared to 1 M NaClO_4_-TEP/VC electrolyte (see Fig. S41). This suggests that the VC-rich solvation structure facilitated by the enhanced ion–ion interactions successfully promotes the formation of anion-/carbonate-derived interfacial chemistry to inhibit the successive decomposition of TEP, thereby forming a robust mosaic-type organic–inorganic hybrid EEI on the electrode surface.

**Figure 5.**
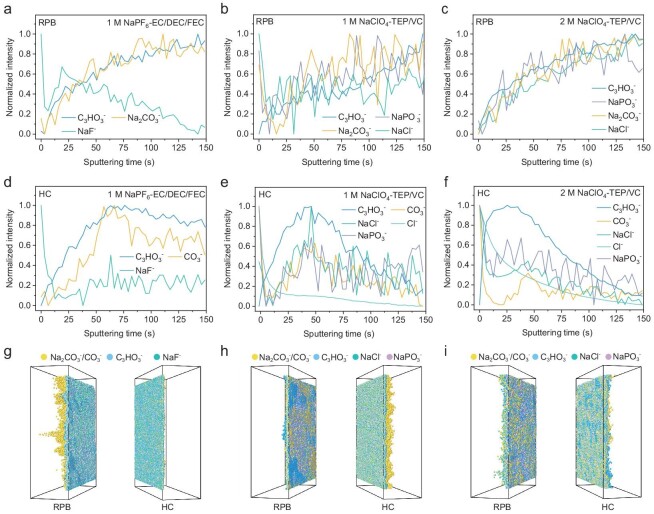
TOF-SIMS depth profiles and the constructed 3D configuration of selected secondary ion fragments on the CEI and SEI. Normalized depth profiling of several typical second ion fragments on the cycled RPB cathodes with (a) 1 M NaPF_6_-EC/DEC/FEC, (b) 1 M NaClO_4_-TEP/VC and (c) 2 M NaClO_4_-TEP/VC electrolyte. Normalized depth profiling of several typical second ion fragments on the cycled HC anodes with (d) 1 M NaPF_6_-EC/DEC/FEC, (e) 1 M NaClO_4_-TEP/VC and (f) 2 M NaClO_4_-TEP/VC electrolyte. Corresponding 3D spatial distribution of CEI and SEI in (g) 1 M NaPF_6_-EC/DEC/FEC, (h) 1 M NaClO_4_-TEP/VC and (i) 2 M NaClO_4_-TEP/VC electrolyte.

In order to further explore the structural and chemical differences of CEI and SEI layers formed in different solvation structures and electrolytes, TOF-SIMS was carried out to visualize the chemical evolution occurring at CEI and SEI. According to the focused ion beam-SEM (FIB-SEM) image, the chemical compositions of passivation film are mainly distributed in the sputtering time of 0–60 s, as is evident from the obvious cracks and underlying electrode materials appearing in the subsequent sputtering time (see Figs S42–S44). As shown in Fig. 5a–f, plenty of secondary ion fragments are obtained. The C_3_HO_3_^−^ fragments are commonly regarded as the organic species of CEI and SEI, which may come from the decomposition of cyclic carbonate including VC, FEC and EC, while the NaPO_3_^−^, Na_2_CO_3_^−^, CO_3_^−^, NaF^−^, NaCl^−^ and Cl^−^ are generally considered as the inorganic species of CEI and SEI. In Fig. 5a, d, the CEI and SEI in 1 M NaPF_6_-EC/DEC/FEC electrolyte composed of an organic–inorganic double layer exhibits different trends of NaF^−^ and C_3_HO_3_^−^ with the deepening of sputtering, which has been reported previously [14]. Fig. 5b, e shows that the content of Na_2_CO_3_^−^, CO_3_^−^, NaCl^−^ and NaPO_3_^−^ drops sharply and then rises during the sputtering time of 0–50 s, while the content of C_3_HO_3_^−^ increases continuously, indicating that a nonuniform CEI and SEI with high internal resistance was formed in 1 M NaClO_4_-TEP/VC due to the poor ionic conductivity of Na_2_CO_3_^−^ and NaPO_3_^−^ species produced from continuous decomposition of TEP. On the contrary, organic and inorganic species on the surface of RPB exhibit an almost identical tendency in 2 M NaClO_4_-TEP/VC electrolyte, and the content of C_3_HO_3_^−^, NaCl^−^ and NaPO_3_^−^ on the HC surface also remain relatively stable during the sputtering time of 5–50 s (Fig. 5c, f), which means that the organic and inorganic species in CEI and SEI layers are evenly distributed. In addition, stronger C^−^, O^−^ and Cl^−^ signals are also discovered in the inside of CEI and SEI formed in 2 M NaClO_4_-TEP/VC electrolyte, which corresponds to the mixed decomposition products of VC and ClO_4_^−^ (see Figs S45–S51). The corresponding 3D reconstructions of CEI and SEI layers directly visualize the spatial distribution of organic and inorganic components are shown in Fig. 5g–i. The CEI and SEI exhibit a dual-layer structure composed of a C_3_HO_3_^−^-rich organic outer layer and a Na_2_CO_3_^−^/NaF^−^-rich inorganic inner layer in 1 M NaPF_6_-EC/DEC/FEC electrolyte. However, the CEI and SEI exhibit uniform distribution of mixed organic and inorganic species with a mosaic-type monolayer structure in 2 M NaClO_4_-TEP/VC electrolyte, which is consistent with the previous cryo-TEM and XPS analysis. In contrast, the products of CEI in 1 M NaClO_4_-TEP/VC electrolyte show a highly heterogeneous distribution, in which there is a clear boundary between organic and inorganic components, while the SEI is dominated by a high content of Na_2_CO_3_^−^ and NaPO_3_^−^, which is not seen in the 2 M NaClO_4_-TEP/VC electrolyte (see Figs S52 and S53). As a result, the 2 M NaClO_4_-TEP/VC electrolyte demonstrates promising interfacial chemistry dominated by anion and additive co-decomposition, which delivers improved stability against the anode and cathode.

## CONCLUSION

In summary, we successfully developed a fluorine-free non-flammable electrolyte for low-cost, safe, environmentally friendly and stable sodium-ion batteries through an ion–ion/ion–dipole interaction modulation strategy. This unique solvation structure is enabled by reorganizing the ligands in the Na^+^ solvation sheath via the regulation of ion–ion and ion–dipole interactions between Na^+^ and anions, carbonate and phosphonate molecules. This precise manipulation of the Na^+^ primary solvation sheath facilitates an anion-/carbonate-rich solvation structure, which leads to the formation of an anion-/carbonate-derived interfacial chemistry. The experimental evidence of XPS, cryo-TEM and TOF-SIMS proved that the architecture of CEI and SEI changed from a monolithic-type structure to a mosaic-type structure when the Na^+^ solvation structure changed from a phosphonate-rich to anion-/carbonate-rich solvation sheath, suggesting that the adverse decomposition of solvents is inhibited. Using this design, a 2 M NaClO_4_-TEP/VC fluorine-free non-flammable electrolyte was identified, a fluorine-free RPB||HC pouch cell with 2 M NaClO_4_-TEP/VC electrolyte displayed excellent cycling stability (96.0% capacity retention after 50 cycles) and high energy density (221.7 Wh kg^−1^ based on electrode mass and 115.1 Wh kg^−1^ based on total battery mass) with enhanced safety performance. Meanwhile, this electrolyte design method has also been extended to other cyclic carbonates. This study suggests a way to overcome the poor compatibility of phosphonate-based electrolytes, thereby enabling the presence of a higher proportion of phosphates in electrolyte systems. Our results also provide insights into understanding the CEI and SEI formation process in full cells and tuning its chemical composition, which is critical for guiding the fabrication of sodium-ion batteries with high performance and practical application.

## Supplementary Material

nwae466_Supplemental_Files
