# Supplementary material for: Modulating ion–dipole interactions in nonflammable phosphonate-based electrolyte for safe and stable sodium-ion pouch cells
Source: Natl Sci Rev. 2024 Dec 23;12(3):nwae466. doi: 10.1093/nsr/nwae466 (PMC11843547; doi:10.1093/nsr/nwae466)
Supplement: nwae466_Supplemental_Files [file nwae466_supplemental_files.zip › Supporting_Information.pdf]

Supporting Information

Modulating Ion-Dipole Interactions in Nonflammable Phosphonate-Based Electrolyte for Safe and Stable Sodium-Ion Pouch Cells

Zhuo Yang[1,2,3], Yingying Dai[1], Zheng-Kun Xie[4], Shao-Bo Li [5], Yao-Jie Lei [3], Jian Chen [6], Xunzhu Zhou [1,6], Zhi-Qiang Hao[1,6], Xin Tan[1,6], Lin Li[1,6], Wei-Hong Lai[2]*, Li Li[7]*, Wei-Hua Chen[4] and Shu-Lei Chou[1,6]**

1. Institute for Carbon Neutralization Technology, College of Chemistry and Materials Engineering, Wenzhou University, Wenzhou, Zhejiang 325035, P.R. China
   E-mail: linli@wzu.edu.cn; chou@wzu.edu.cn

2. Laboratory of Advanced Materials, Shanghai Key Lab of Molecular Catalysis and Innovative Materials, Fudan University, Shanghai, 200438, P.R. China
   E-mail: weihongl@fudan.edu.cn

3. Institute for Superconducting and Electronic Materials University of Wollongong, Innovation Campus, Squires Way, Wollongong, New South Wales 2500, Australia

4. College of Chemistry, Zhengzhou University, Zhengzhou, Henan 450001, P. R. China

5. School of Materials Science and Engineering, South China University of Technology, Guangzhou 510640, P.R. China

6. Wenzhou Key Laboratory of Sodium-Ion Batteries, Wenzhou University Technology Innovation Institute for Carbon Neutralization, Wenzhou, Zhejiang 325035, P.R. China

7. School of Environmental and Chemical Engineering, Shanghai University, Shanghai 200444, China
   E-mail: lili2020@shu.edu.cn;

Materials and Methods

Materials. PC, FEC were purchased from Sigma Aldrich and used after being dehydrated with a 4-Å molecular sieve. $NaPF_6$ purchased from DoDochem Technology, was dissolved into PC with the addition of 5%wt FEC with the salt concentration of 1 M in a glovebox filled with argon. The solution of 1 M $NaPF_6$ in PC was used as received as a comparison. EC, PC, DEC, EMC, FEC, VC and TEP were purchased from Sigma Aldrich and used after being dehydrated with a 4-Å molecular sieve. $NaClO_4$ purchased from Aladdin Biochemical Technology, was dissolved into TEP with the addition of 5%wt VC in predetermined concentrations in a glovebox filled with argon. The solution of 1 M $NaPF_6$ in EC/DEC with 5%wt FEC was used as received as a comparison. The rhombohedral $Na_{2-x}FeFe(CN)_6$ (RPB) obtained from monoclinic $Na_{2-x}FeFe(CN)_6$ by a simple heat treatment step. The monoclinic Prussian blue of $Na_{2-x}FeFe(CN)_6$ was synthesized by a precipitation methods at 25 ℃.[1] Two mixtures, sodium citrate and $FeSO_4 \cdot 7H_2O$ (A) (from Aladdin Biochemical Technology), and sodium citrate and $Na_4Fe(CN)_6 \cdot 10H_2O$ (B) (from Aladdin Biochemical Technology), were dissolved in deionized water, respectively. Both of solutions were bubbled with N_2 to protect Fe^{2+} from oxidation. Then, solution A was added dropwise into solution B, and the mixture (A+B) was kept stirring at 800 rpm for 6 h and aged for 2 hours before centrifugation. The precipitate was washed with deionized water and ethanol three times, respectively. Finally, the monoclinic Prussian blue powder was converted RPB powder after drying in a vacuum oven at 180 ℃ for 24 h (Figure S54, Supporting Information). The hard carbon was commercially purchased from KURARAY and used as-received. The separator used in this study was a cellulose separator with 25 μm, purchased from NIPPON KODOSHI.

Fabrication of cells. All 2032-type coin and pouch cells were assembled in with cellulose separator in an Ar-filled glove box (O_2 and H_2O content < 0.1 ppm). RPB and HC electrodes for coin cell were prepared by mixing active materials with carbon black and polyvinylidene fluoride (PVDF) in a weight ratio of 90:5:5 using N-methyl pyrrolidinone (NMP) as the solvent and were cast onto an aluminum foil by coating machine, the RPB and HC electrode dried at 150 °C and 100 °C for 24 h under vacuum, respectively. The electrodes were punched into discs with diameters of 14 mm and 12 mm for anodes and cathodes, respectively. The 60 μl electrolyte and a piece of cellulose separator were employed to fabricate a coin cell. In the case of pouch cell, we used sodium alginate (SA) as the binders to build fluoride-free sodium-ion batteries. The electrode was fabricated by mixing the active materials, SA, and carbon black with the weight ratios of 90:5:5 the in deionized water, then the slurries were

cast on aluminum foil by coating machine. Finally, all the electrodes for pouch cell were stored in an Ar-filled glove box after drying in a vacuum oven at 180 ℃ for 24 h.

Electrochemical measurements. The performance of these cells was tested on a Neware battery test system (CT4008T-5V12A and CT4008T-5V10mA, Shenzhen, China). The cycling and rate performances of RPB||HC coin cells were measured at 25 ℃ with a voltage range from 1.5 to 3.4 V. The cycling performances of RPB||HC pouch cells were measured at 25 °C with a voltage range from 1.5 to 3.6 V for first several cycles and 1.5 to 3.4 V for subsequent cycles. The cyclic voltammetry (CV), linear sweep voltammetry (LSV) and electrochemical impedance spectroscopy (EIS) measurements were performed on a CHI600C Electrochemical Analytical Instrument (Chenhua). The cyclic CV measurements of RPB||HC cells were examined between 1.5 and 3.6 V at a scanning rate of 0.05 mV s^{-1} under 25 °C. The LSV measurements of Na||stainless steel and Na||Al coin cells with different electrolytes were examined from 2.5 to 6 V vs. Na at a scanning rate of 0.5 mV s^{-1}. The EIS were tested over the frequency range of 0.01-10,000 Hz.

Characterization. The ionic conductivity and wettability of the electrolytes were determined with a conductivity measuring meter (DDS-307, Leici) and a Contact Angle Meter (JY-82B Kruss DSA), respectively. Thermogravimetry (TG) was performed with a Mettler-Toledo instrument at a ramping rate of 10 ℃ min^{-1}. Closed-cup flash point was test with MINIFLASH FP VISION-Grabner. SEM characterizations were conducted with a Zeiss Sigma 300, The XPS results on the CEI and SEI compositions were obtained using a Thermo Scientific K-Alpha with Al Kα radiation (pass energy 55.0 eV) at a pressure lower than 2×10^{-7} Torr. Cyro-TEM was performed using FEI Krios (USA) operated at 300 kV, which is equipped with automatic injection system of frozen sample. The sample preparation process operated under Ar atmosphere, and TEM grids is taken out from centrifuge tube and transferred to the TEM column in the liquid N_2. Time-of-Flight Secondary Ion Mass Spectrometry (TOF-SIMS) measurements were conducted in the positive and negative mode (TESCAN MAIA3). A pulsed 30 keV Ga^+ (3nA) ion beam was used on the electrode. The TOF-SIMS measurements were carried out over an electrode sample volume with a 30 μm × 30 μm sputtering area times an around 2 μm sputtered depth for an electrode surface that was selected after full cell discharge to 1.5 V. These samples used for interphase characterization in 1 M $NaPF_6$-EC/DEC electrolyte and other electrode samples (1 M $NaClO_4$-TEP/VC, 2 M $NaClO_4$-TEP/VC) were washed three times with DEC and TEP, respectively. And these samples were loaded into the tester chamber in an airtight vessel without exposure to the ambient air. Raman spectra of all the electrolytes were collected using a LabRAM HR

Evolution instrument with a 632.8 nm excitation laser. The ^{17}O solid-state NMR spectra of the electrolytes were recorded on a 600 MHz WB Solid-State Nuclear Magnetic Resonance system (Bruker AVANCE NEO 600 WB) at room temperature, and no deuterated reagents were used.

Computational methods. Quantum chemistry calculations were carried out with the Gaussian 16 software package. The structure optimization, molecular orbital energy as well as binding energy calculation were performed with the B3LYP density functional and the 6-311g(d,p) basis set.[2,3] The SMD solvation model was used to describe the solvation effect. Acetone was used as the solvent for calculation of Na^+ complexes. MD simulations for the electrolyte structures were conducted by using the GROMACS package with AMBER03 force field.[4,5] 100 $NaClO_4$ and 480 TEP (1 M $NaClO_4$-TEP), 200 $NaClO_4$ and 570 TEP (2 M $NaClO_4$-TEP), 100 $NaClO_4$, 450 TEP and 30 VC (1 M $NaClO_4$-TEP/VC), 200 $NaClO_4$, 500 VC and 70 TEP (2 M $NaClO_4$-TEP/VC), 100 $NaClO_4$ and 480 VC (1 M $NaClO_4$-VC), 200 $NaClO_4$, 570 VC (2 M $NaClO_4$-VC), 100 $NaClO_4$, 450 VC and 30 TEP (1 M $NaClO_4$-VC/TEP), and 200 $NaClO_4$, 500 VC and 70 TEP (2 M $NaClO_4$-VC/TEP) were set up randomly in a cubic simulation box. The MD parameters for Na^+ were in the built-in force field parameters. The MD parameters for $NaClO_4$, TEP, and VC were generated by ACPYPE.[5] The atomic charges of solvent were corrected by a factor of 1.5. Upon quasi-equilibrium of the system, a total of 90 ns MD simulation was carried out for each electrolyte. First, NVT runs were performed at 398.15 K for 40 ns, and then NPT runs of 40 ns were performed at 298.15 K to ensure the system equilibrium. Finally, a 10 ns NPT runs were used for calculating the radial distribution functions and coordination numbers in various electrolytes. The snapshot of MD simulation is produced by VMD software.

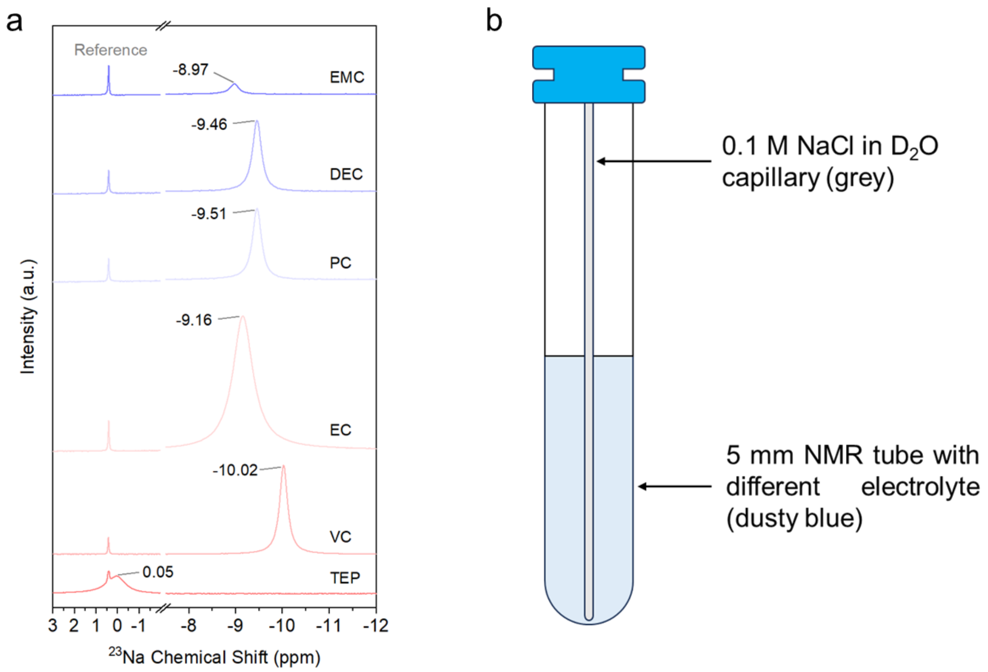

Supplementary Figure S1 (a) The ^{23}Na NMR chemical shifts of the electrolytes with different solvents. The salt concentration of $NaClO_4$ in all electrolytes is 0.1 M. (b) Schematic of test sample, insert a capillary containing 0.1 M NaCl in H_2O electrolyte into the NMR tube for reference.

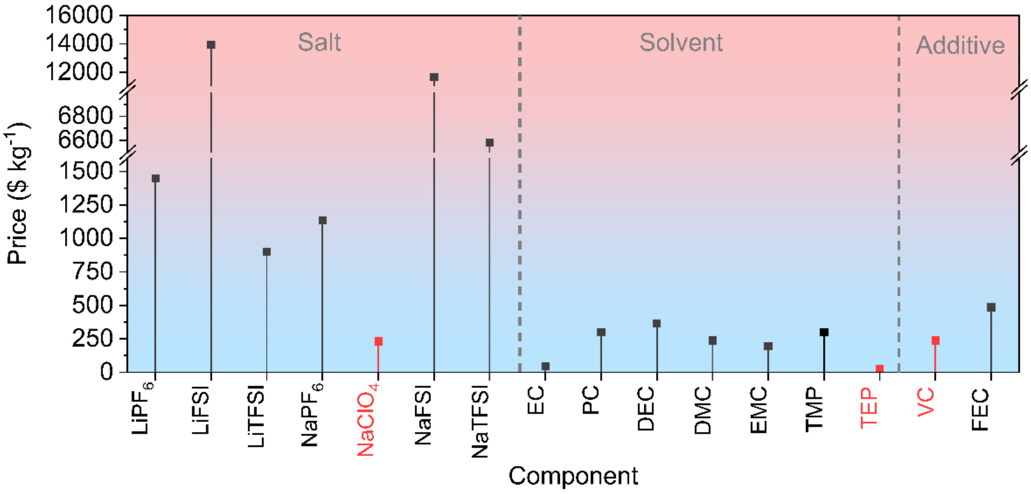

Supplementary Figure S2 Cost comparison for commonly used Li/Na salts, solvents and additives (price is from Reaxys, last updated on 22nd Oct 2023).

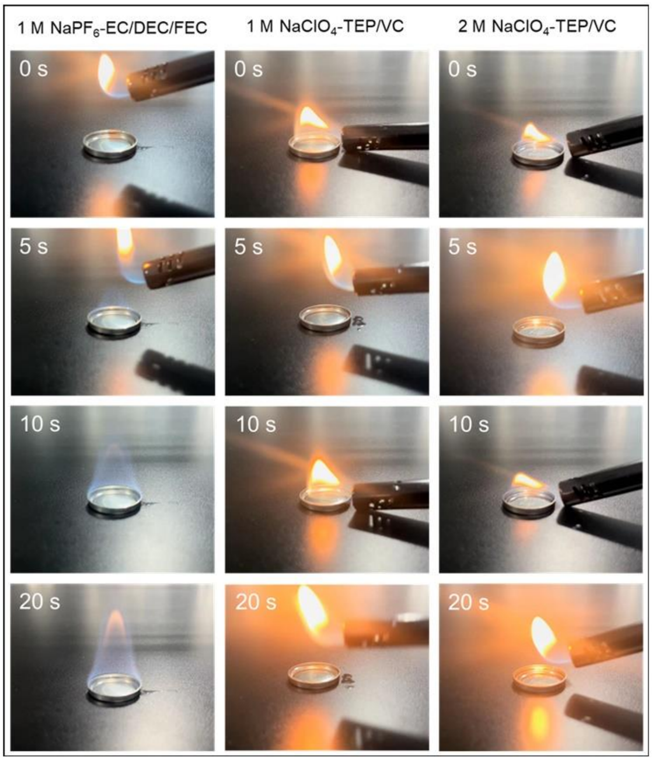

Supplementary Figure S3 Flame tests of 1 M $NaPF_6$-EC/DEC/FEC, 1 M $NaClO_4$-TEP/VC and 2 M $NaClO_4$-TEP/VC electrolyte.

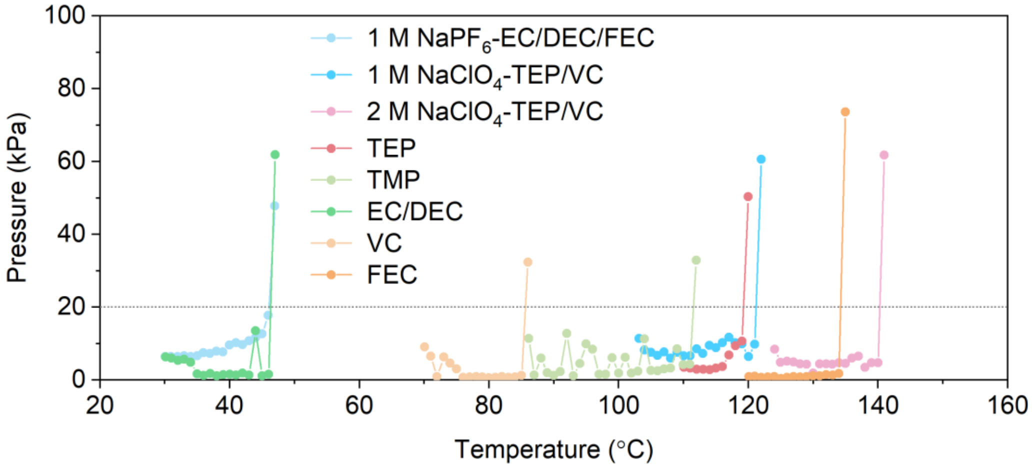

Supplementary Figure S4 Comparison of the closed-cup flash point for three different electrolytes and their components.

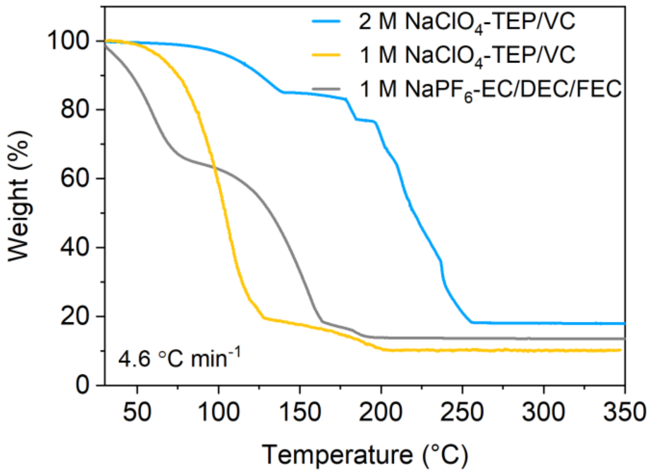

Supplementary Figure S5 Weight loss of 1 M $NaPF_6$-EC/DEC/FEC, 1 M $NaClO_4$-TEP/VC and 2 M $NaClO_4$-TEP/VC electrolytes in the thermogravimetric test.

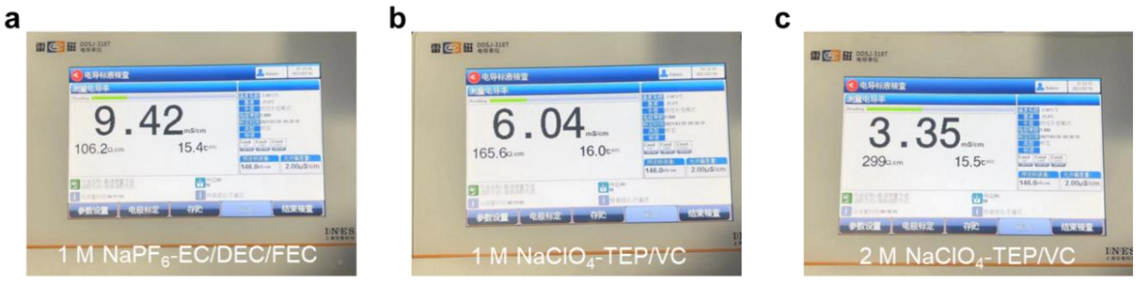

Supplementary Figure S6 Ionic conductivity of (a) 1 M $NaPF_6$-EC/DEC/FEC, (b) 1 M $NaClO_4$-TEP/VC and (c) 2 M $NaClO_4$-TEP/VC electrolyte.

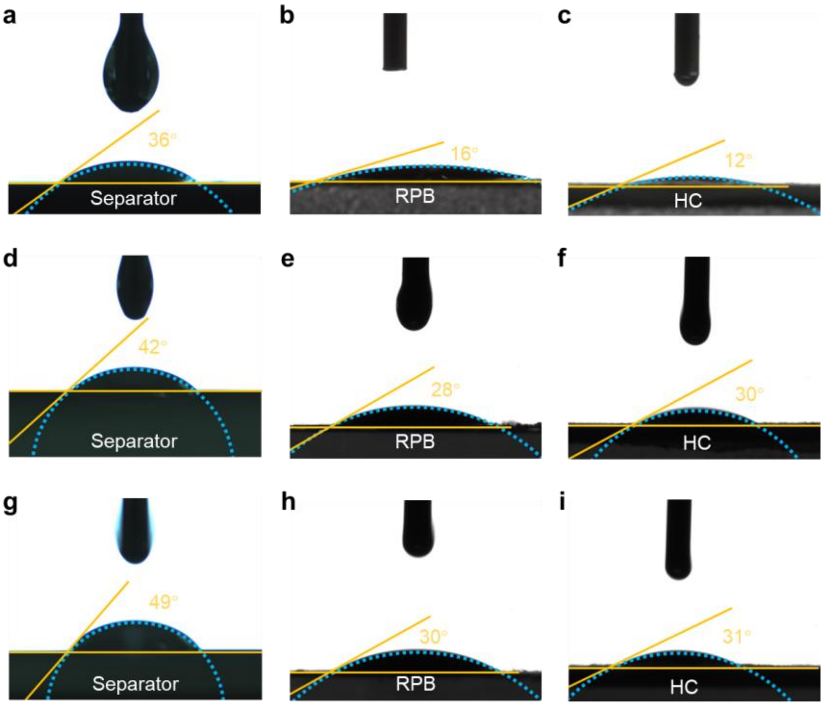

Supplementary Figure S7 Wettability tests of separator, RPB cathode and HC anode in contact with (a-c) 1 M $NaPF_6$-EC/DEC/FEC, (d-f) 1 M $NaClO_4$-TEP/VC and (g-i) 2 M $NaClO_4$-TEP/VC electrolyte.

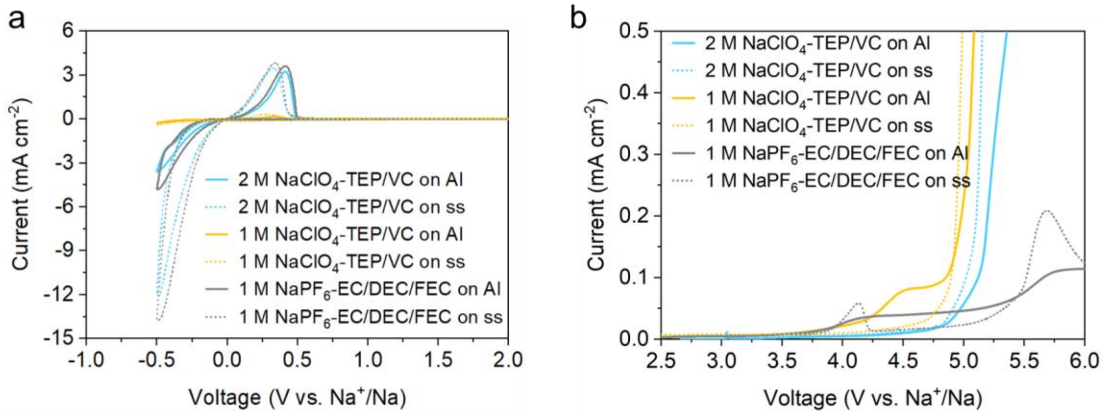

Supplementary Figure S8 CV curves and linear sweep voltammetry (LSV) profiles of Na||Al and Na||stainless steel (SS) cells with different electrolytes from (a) -0.5 V to 2.0 V and (b) 2.5 V to 6.0 V at a scanning rate of 5 mV s^{-1}, respectively.

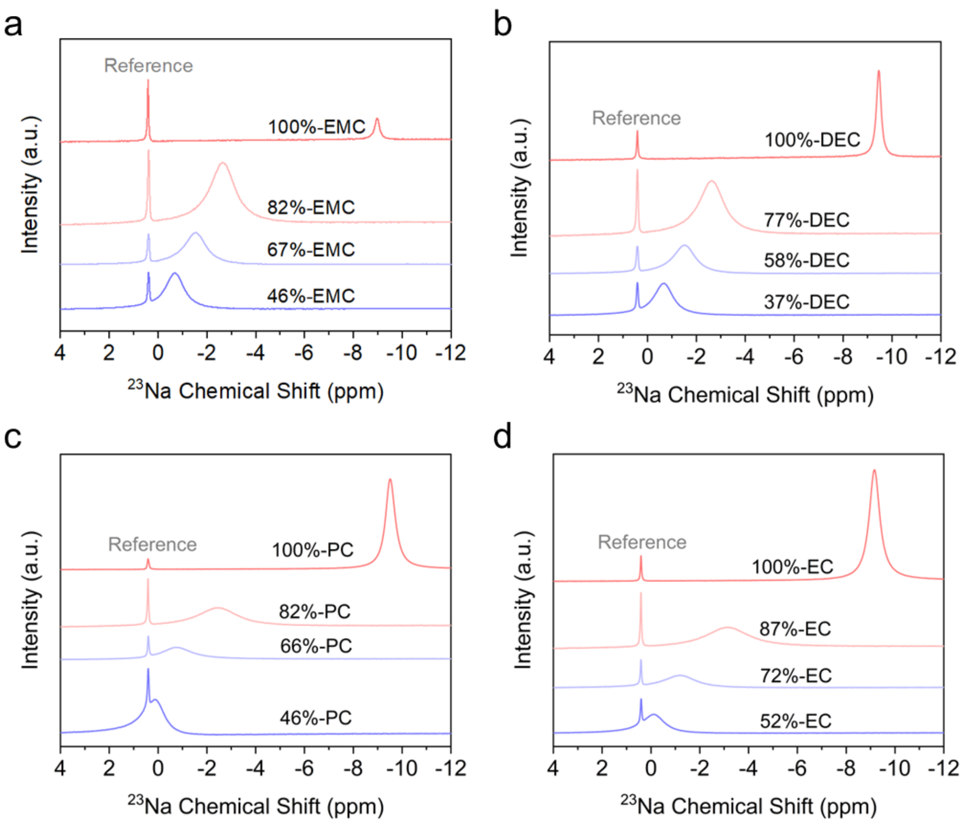

Supplementary Figure S9 The displacement of ^{23}Na NMR chemical shifts with the change of (a) EMC, (b) DEC, (c) PC and (d) EC content.

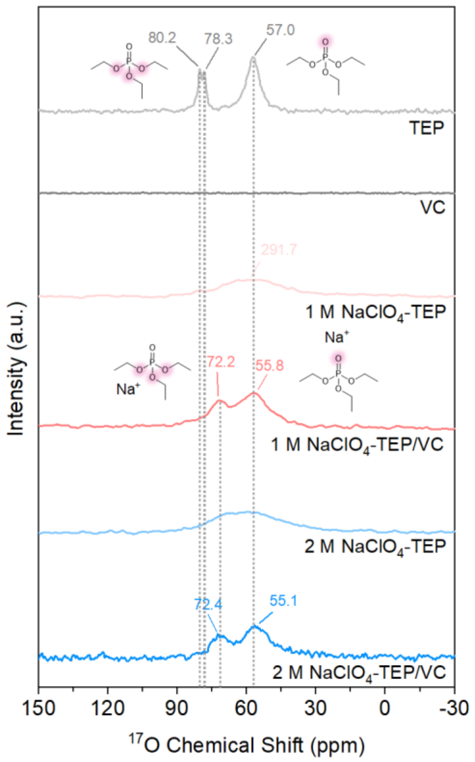

Supplementary Figure S10 ^{17}O-NMR spectra of the different components and electrolytes.

Discussion: In pure TEP solvent, two broad peaks can be easily identified at near 57 and 80 ppm, corresponding to the P=O and P-O of TEP, respectively. With the introduction of $NaClO_4$, the ^{17}O signals of P=O and P-O in TEP were merged into a single broadened signal in both 1 M $NaClO_4$-TEP and 2 M $NaClO_4$-TEP electrolyte due to the deshielding effect caused by interactions between Na^+ and TEP, suggesting the formation of TEP-dominated solvation structure. Nevertheless, with the addition of VC in 1 M $NaClO_4$-TEP and 2 M $NaClO_4$-TEP electrolyte, the merged P=O/P-O peak split into two separate peaks. This observation suggests that the addition of VC replaces partially coordinated TEP, thereby attenuating the overall interaction of Na^+-TEP interactions and leading to a reduction in the deshielding effect around Na^+-TEP. More importantly, the ^{17}O signals of C=O in VC showed a chemical shift from 175.4 to 174.1 ppm in 1 M $NaClO_4$-TEP/VC electrolyte but disappeared completely in 2 M $NaClO_4$-TEP/VC electrolyte. This indicates the increased amount of VC molecules coordinating with Na^+ and stronger interactions between Na^+ and VC in the 2 M $NaClO_4$-TEP/VC electrolyte, leading to the complete disappearance of C=O signal caused by a pronounced deshielding effect. The reconfiguration of Na^+ solvation sheath in 2 M $NaClO_4$-TEP/VC electrolyte can also be confirmed by the shift of the C-O signals in VC moves from 219.0 to 218.3 ppm, which indicates that their electron cloud density on the C-O oxygen had

increased, namely, the interaction between Na^+ and VC was enhanced to form a VC-rich solvation structure.

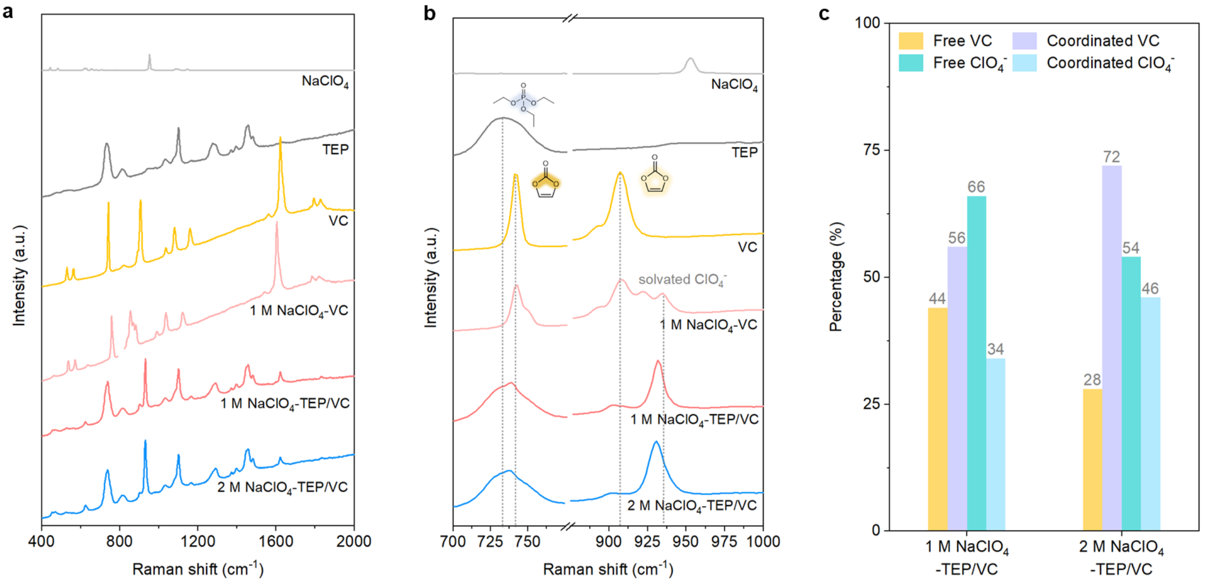

Supplementary Figure S11 (a, b) Full Raman spectra of the corresponding components and electrolytes. (c) The percentages of free/coordinated VC and ClO_4^- in the 1 M $NaClO_4$-TEP/VC and 2 M $NaClO_4$-TEP/VC electrolytes.

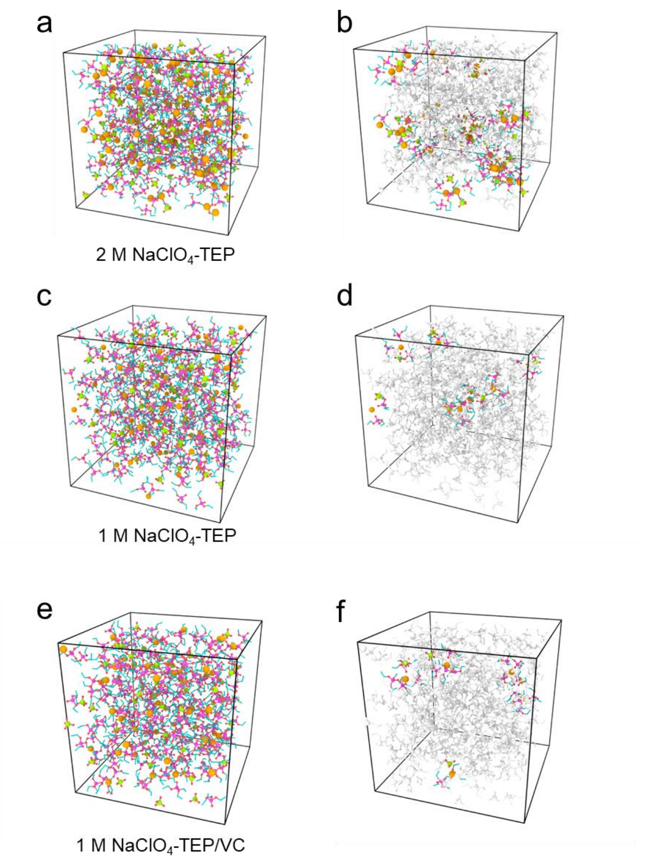

Supplementary Figure S12 Snapshots of the MD simulation boxes of (a) 2 M $NaClO_4$-TEP electrolyte and (b) corresponding aggregate/CIP solvation structure, (c) 1 M $NaClO_4$-TEP electrolyte and (d) corresponding aggregate/CIP solvation structure, (e) 1 M $NaClO_4$-TEP/VC electrolyte and (f) corresponding aggregate/CIP solvation structure.

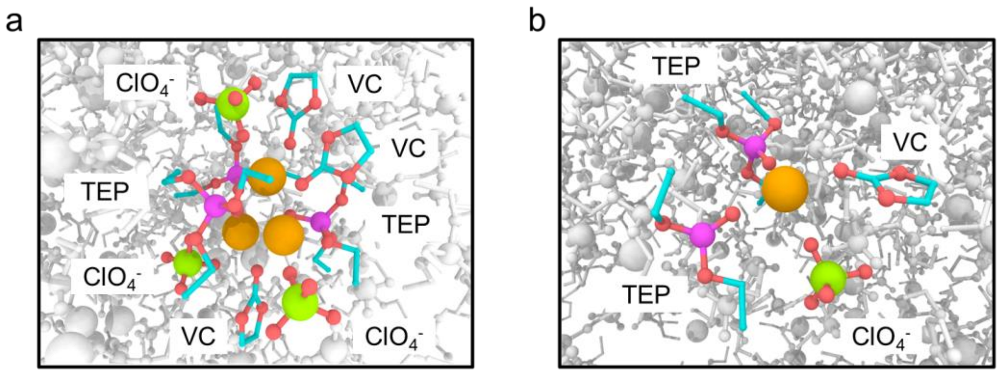

Supplementary Figure S13 Representative solvation structure (i) aggregate and (j) CIP of 2 M $NaClO_4$-TEP/VC electrolyte.

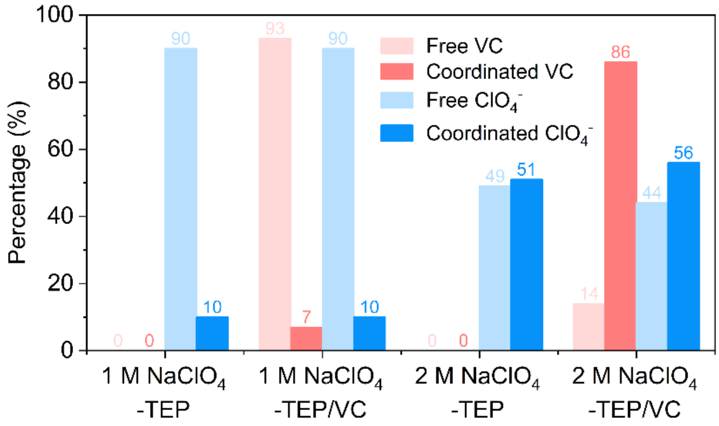

Supplementary Figure S14 The proportions of free VC, coordinated VC, free ClO_4^- and coordinated ClO_4^- in each electrolyte.

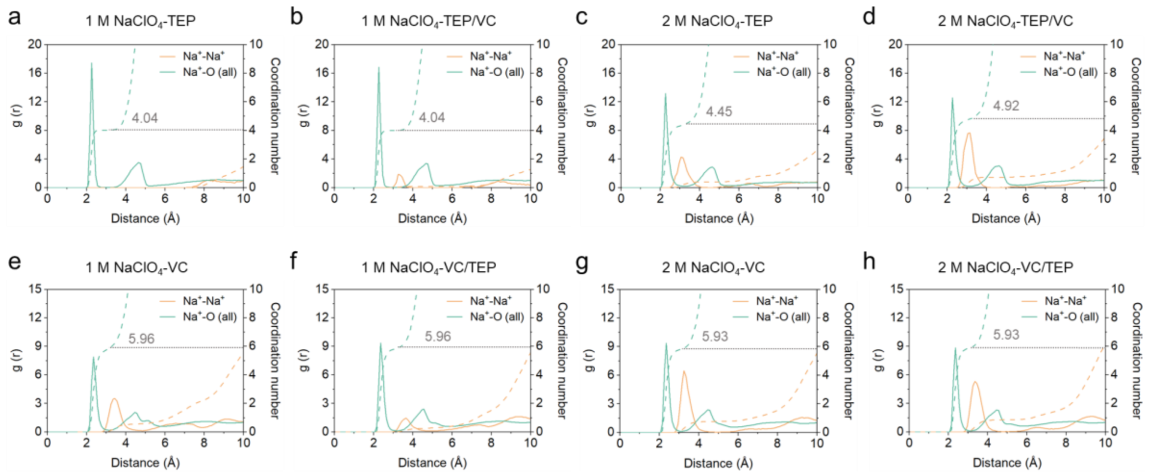

Supplementary Figure S15 The total radial distribution function of (a) 1 M $NaClO_4$-TEP electrolyte, (b) 1 M $NaClO_4$-TEP/VC electrolyte, (c) 2 M $NaClO_4$-TEP electrolyte, (d) 2 M $NaClO_4$-TEP/VC electrolyte, (e) 1 M $NaClO_4$-VC electrolyte, (f) 1 M $NaClO_4$-VC/TEP electrolyte, (g) 2 M $NaClO_4$-VC electrolyte and (h) 2 M $NaClO_4$-VC/TEP electrolyte. The TEP/VC denotes TEP as the solvent with 5 wt% VC additive, and the VC/TEP denotes VC as the solvent with 5 wt% TEP additive.

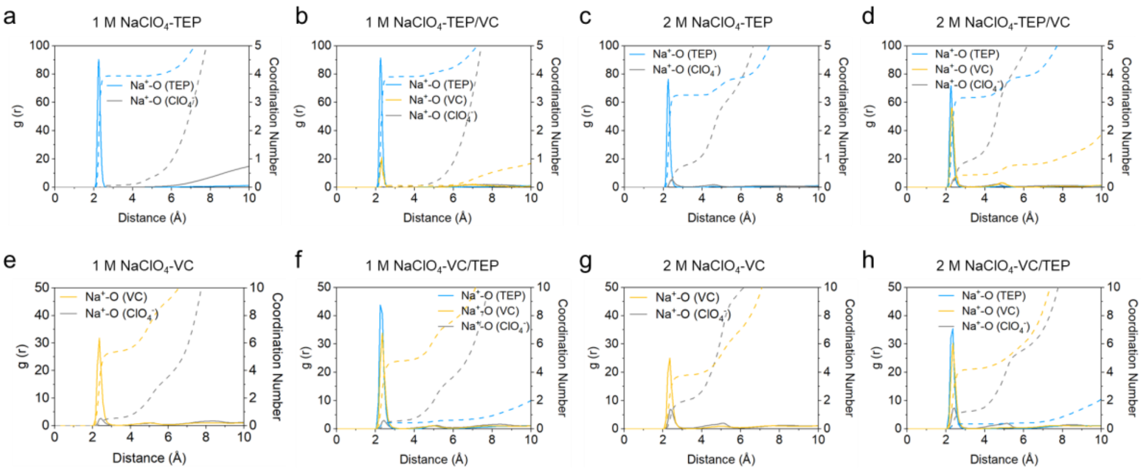

Supplementary Figure S16 The Na-O radial distribution function of (a) 1 M $NaClO_4$-TEP electrolyte, (b) 1 M $NaClO_4$-TEP/VC electrolyte, (c) 2 M $NaClO_4$-TEP electrolyte, (d) 2 M $NaClO_4$-TEP/VC electrolyte, (e) 1 M $NaClO_4$-VC electrolyte, (f) 1 M $NaClO_4$-VC/TEP electrolyte, (g) 2 M $NaClO_4$-VC electrolyte and (h) 2 M $NaClO_4$-VC/TEP electrolyte. The TEP/VC denotes TEP as the solvent with 5 wt% VC additive, and the VC/TEP denotes VC as the solvent with 5 wt% TEP additive.

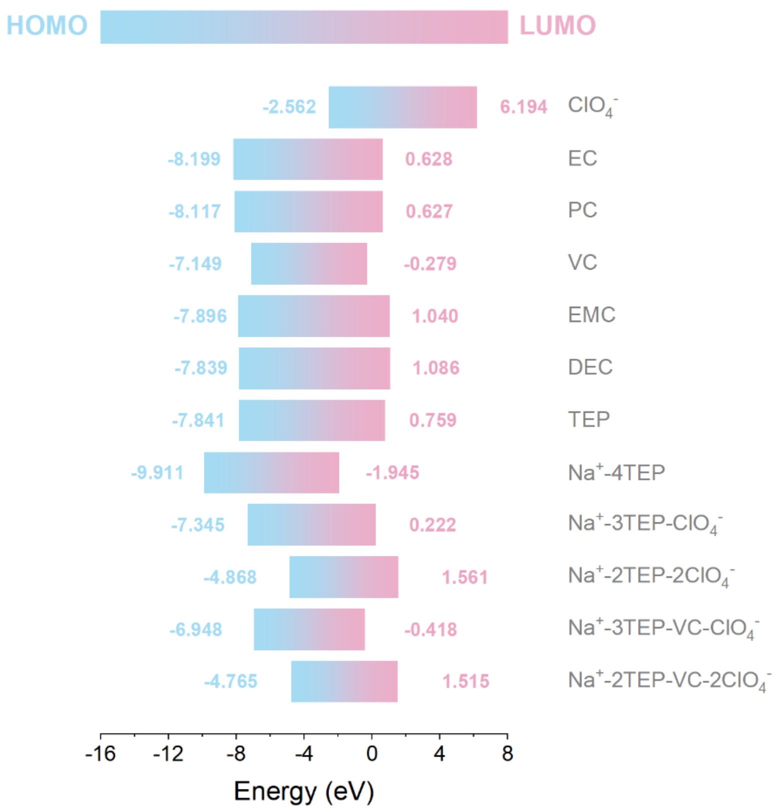

Supplementary Figure S17 The comparison of LUMO-HOMO energy levels for different solvation structure and its components calculated by DFT calculations.

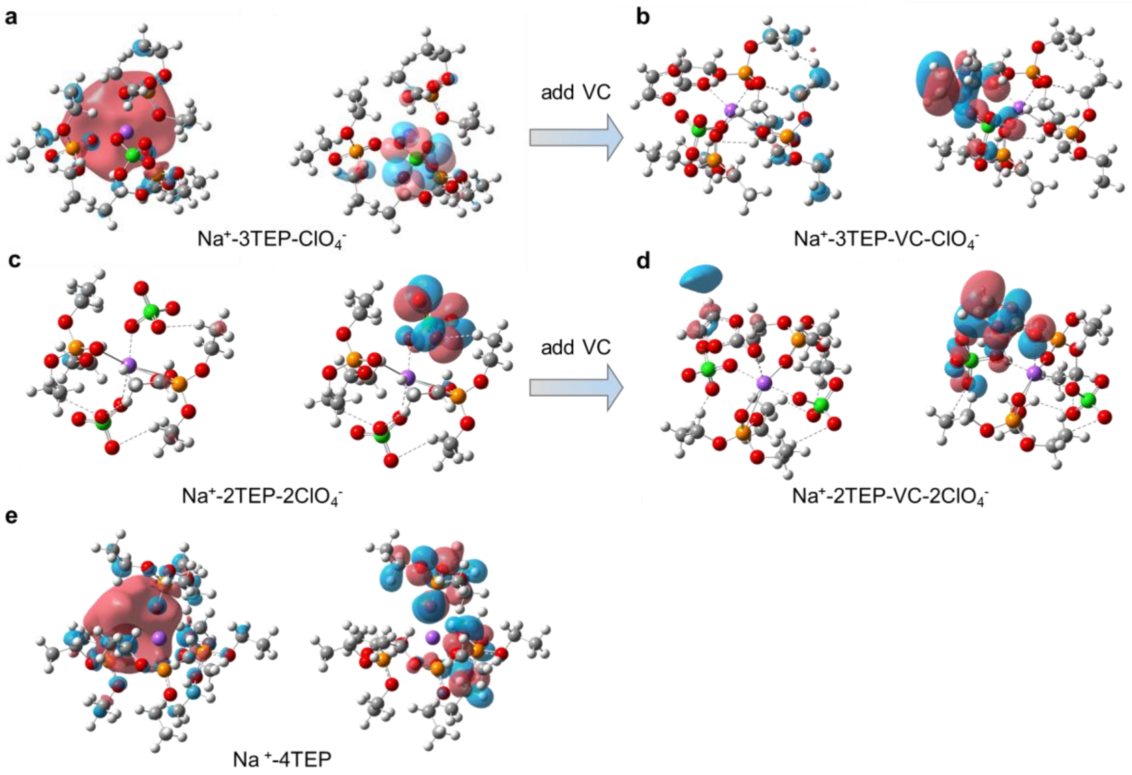

Supplementary Figure S18 The optimized representative Na^+ solvation structure and its LUMO-HOMO in various electrolyte using DFT simulations. (Na-purple, O-red, P-orange, C-gray, Cl-green, H-white).

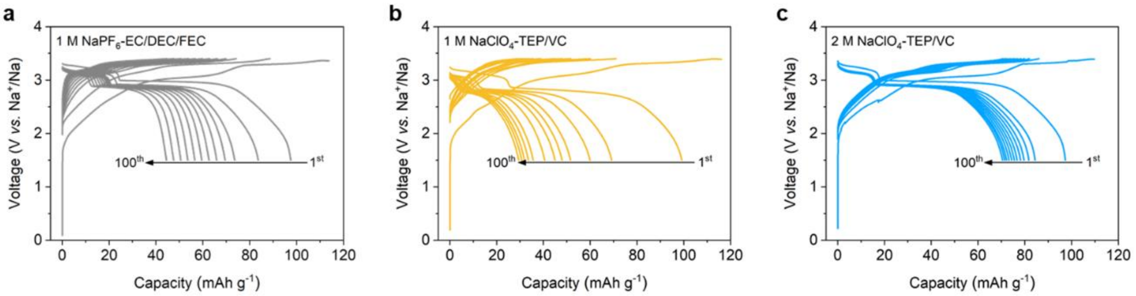

Supplementary Figure S19 Voltage profiles of the RPB||HC coin cells using (a) 1 M $NaPF_6$-EC/DEC/FEC, (b) 1 M $NaClO_4$-TEP/VC and (c) 2 M $NaClO_4$-TEP/VC electrolyte for the 1st to 100th cycles.

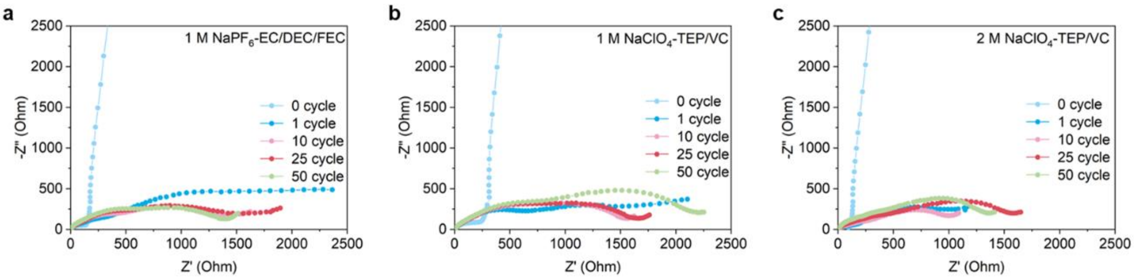

Supplementary Figure S20 Electrochemical impedance spectra for RPB||HC cell in (a) 1 M $NaPF_6$-EC/DEC/FEC, (b) 1 M $NaClO_4$-TEP/VC and (c) 2 M $NaClO_4$-TEP/VC electrolyte after 1st cycle, 10th cycles, 25th and 50th cycles.

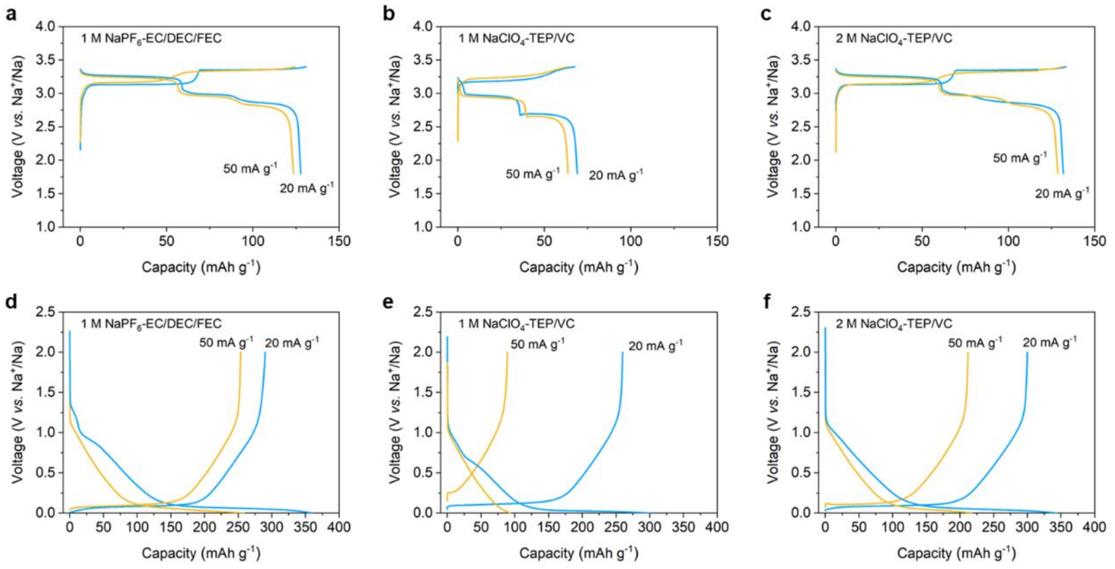

Supplementary Figure S21 Voltage profiles of the RPB||Na and HC||Na half cells at different current density using (a, d) 1 M $NaPF_6$-EC/DEC/FEC, (b, e) 1 M $NaClO_4$-TEP/VC and (c, f) 2 M $NaClO_4$-TEP/VC electrolyte.

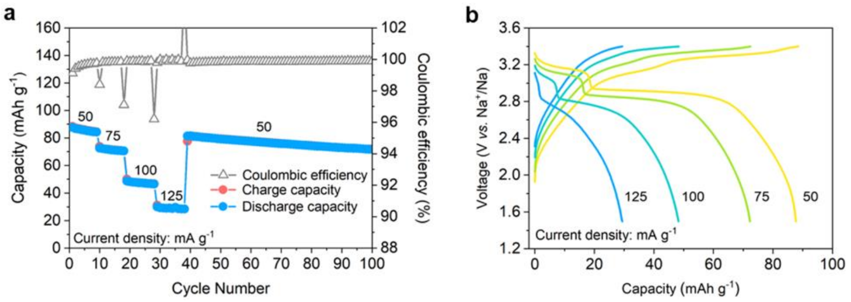

Supplementary Figure S22 (a) Rate performance of the RPB||HC full cells in 2 M $NaClO_4$-TEP/VC electrolyte, and (b) corresponding voltage profiles.

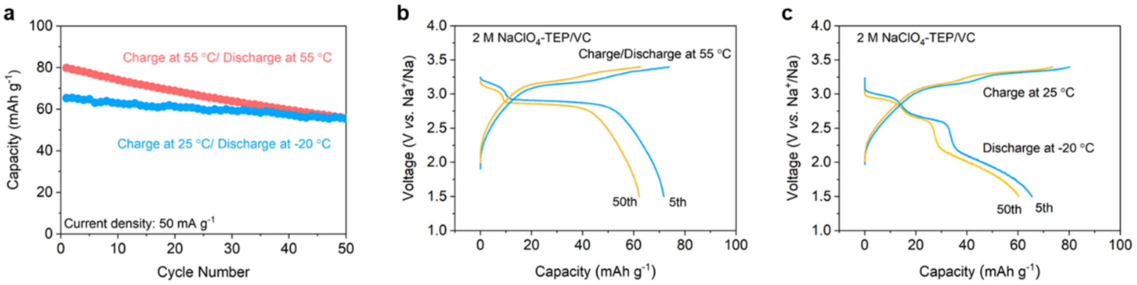

Supplementary Figure S23 (a) The cycling performance of RPB||HC cell with 2 M $NaClO_4$-TEP/VC electrolyte at 55 ℃ and -20 ℃. The corresponding charge-discharge curves at (b) 55 ℃ and (c) -20 ℃.

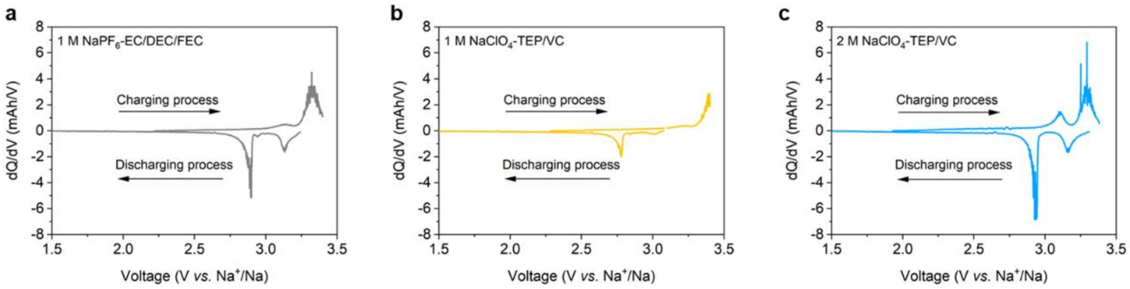

Supplementary Figure S24 Differential capacity analysis of RPB||HC full cells with different electrolytes: (a) 1 M $NaPF_6$-EC/DEC/FEC, (b) 1 M $NaClO_4$-TEP/VC and (c) 2 M $NaClO_4$-TEP/VC electrolyte for second cycles.

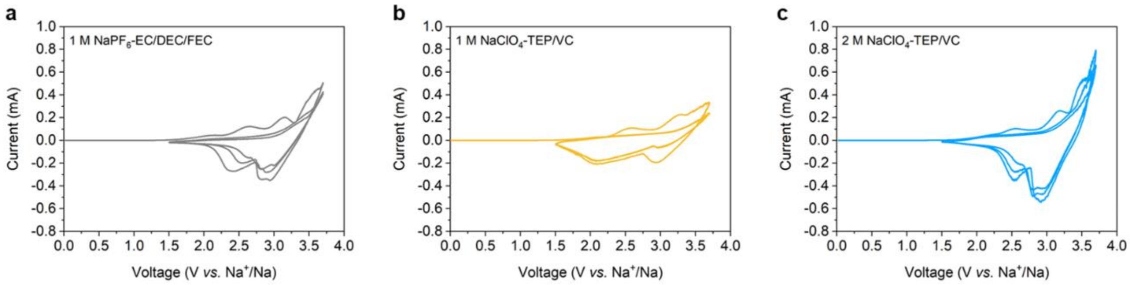

Supplementary Figure S25 CV curves of RPB||HC full cells with different electrolytes: (a) 1 M $NaPF_6$-EC/DEC/FEC, (b) 1 M $NaClO_4$-TEP/VC and (c) 2 M $NaClO_4$-TEP/VC electrolyte for first three cycles.

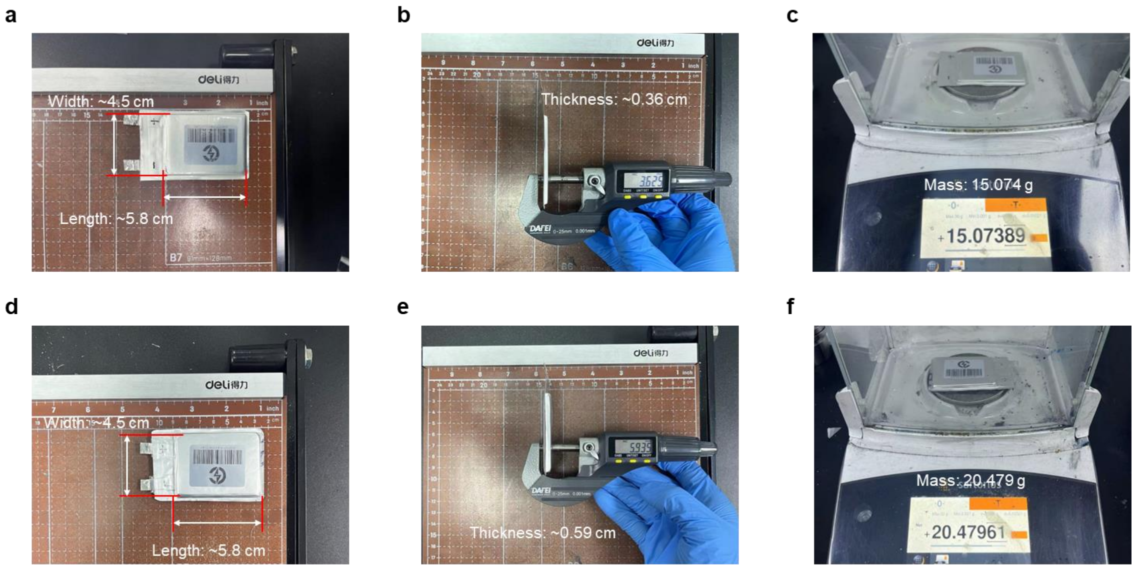

Supplementary Figure S26 The dimensions and practical weight of the pouch cell with (a-c) the cathode mass loading of 10 mg cm^{-2} and anode mass loading of 5 mg cm^{-2}, and (d-f) the cathode mass loading of 20 mg cm^{-2} and anode mass loading of 12 mg cm^{-2}.

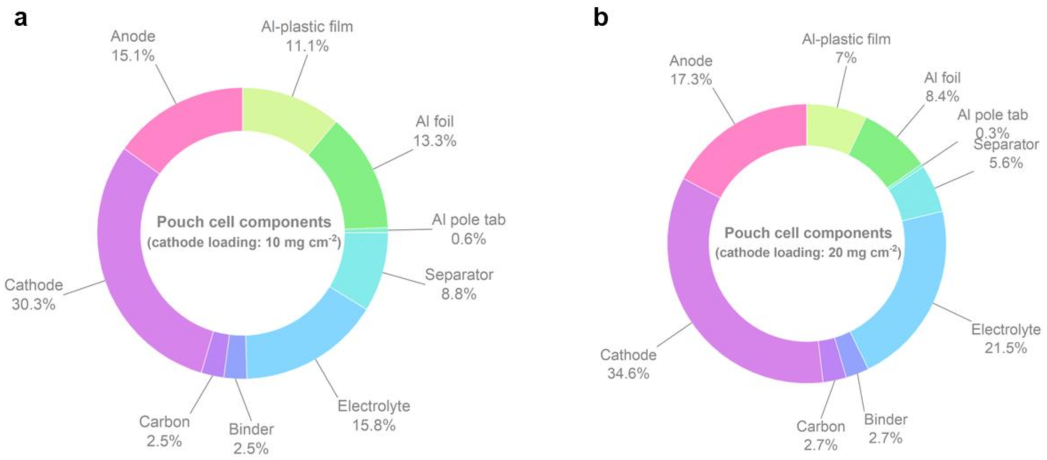

Supplementary Figure S27 Ring plot showing the components and corresponding weight ratios in the pouch cell different mass loading.

Discussion: As the increase of mass loading, there will be a notable increase in the usage of electrolyte, resulting in a greater proportion of battery mass and cost being occupied by the electrolyte. Thus, it is imperative to optimize the property and cost of the electrolyte in order to advance the development of batteries with higher energy density.

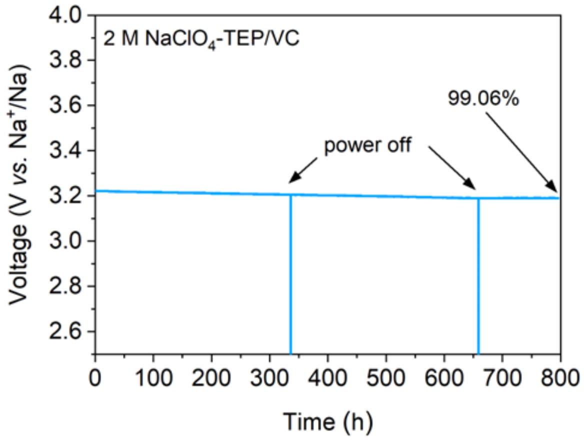

Supplementary Figure S28 The voltage retention rate of RPB||HC pouch cell with 2 M $NaClO_4$-TEP/VC electrolyte.

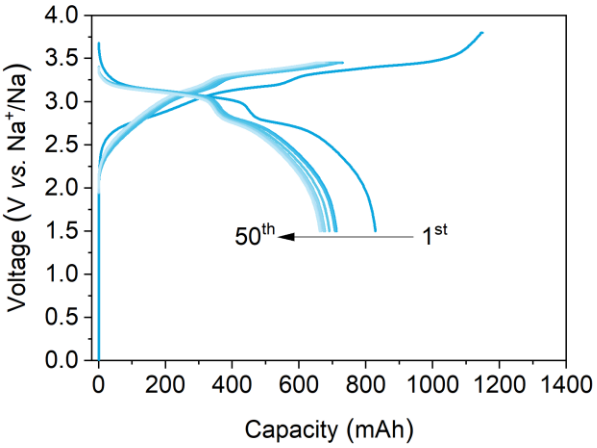

Supplementary Figure S29 Voltage profiles of the RPB||HC pouch cells (cathode mass loading of 20 mg cm^{-2} and anode mass loading of 12 mg cm^{-2}) using 2 M $NaClO_4$-TEP/VC electrolyte for the 1st to 50th cycles.

Discussion: In order to optimize the performance of the RPB||HC pouch cell, we established a cut-off voltage of 3.6 V for initial charging process, and for enhanced cycling performance, we implemented a cut-off voltage of 3.4V in subsequent cycles.

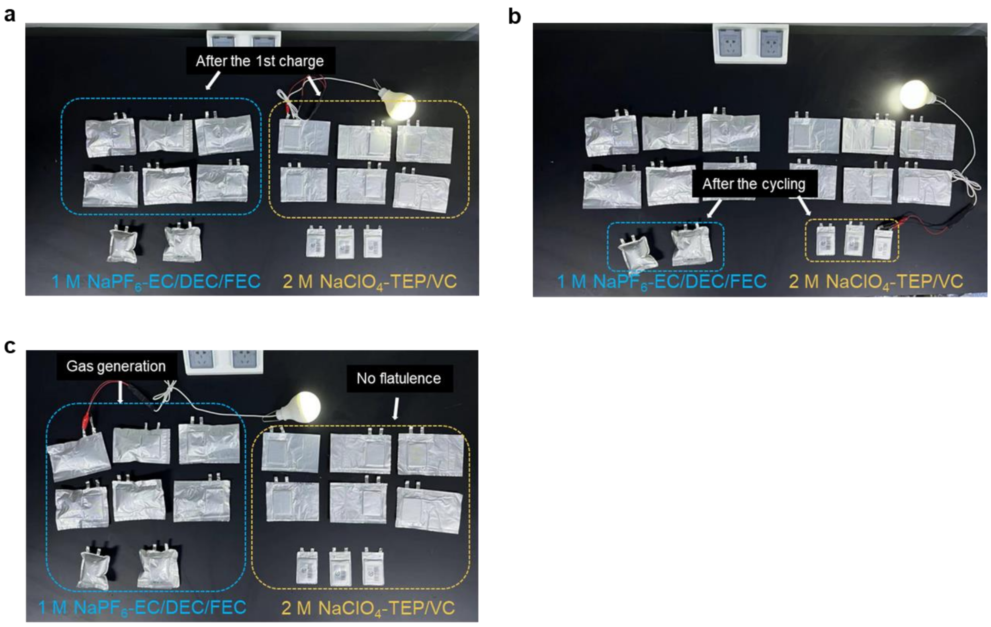

Supplementary Figure S30 Digital photograph of the comparison for gas generation after charge and discharge of RPB||HC pouch cell with 1 M $NaPF_6$-EC/DEC/FEC and 2 M $NaClO_4$-TEP/VC electrolyte.

Discussion: It is evident that the issue of gas generation in carbonate-based electrolytes is of significant concern. Despite implementing exhaust treatment following the initial formation process, substantial gas production persists in subsequent cycles. This occurrence is particularly pronounced in electrodes with higher mass loading, as the increased electrode-electrolyte contact area exacerbates the phenomenon.

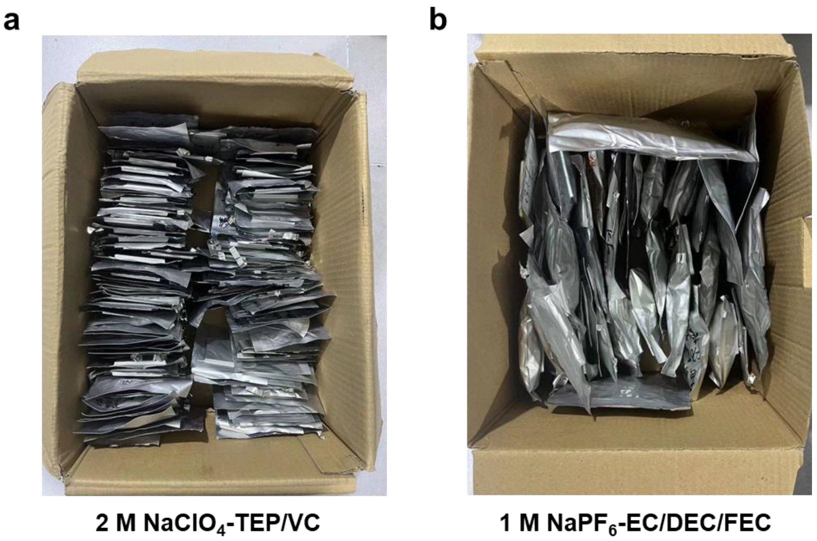

Supplementary Figure S31 Digital photos of the pouch cells we have tested with (a) 2 M $NaClO_4$-TEP/VC electrolyte and (b) 1 M $NaPF_6$-EC/DEC/FEC electrolyte. Almost all pouch cells in 2 M $NaClO_4$-TEP/VC electrolyte exhibited no swelling, but there was obvious gas generation when using 1 M $NaPF_6$-EC/DEC/FEC electrolyte.

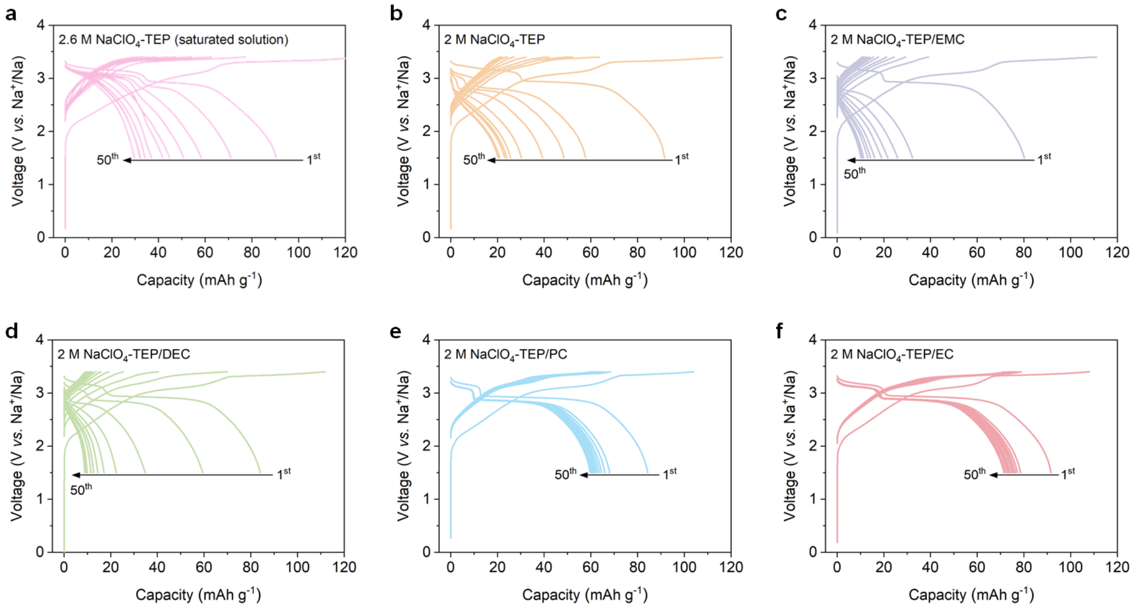

Supplementary Figure S32 Voltage profiles of the RPB||HC coin cells for the 1st to 50th cycles using (a) 2.6 M $NaClO_4$-TEP (saturated solution), (b) 2 M $NaClO_4$-TEP, (c) 2 M $NaClO_4$-TEP/EMC, (d) 2 M $NaClO_4$-TEP/DEC, (e) 2 M $NaClO_4$-TEP/PC and (f) 2 M $NaClO_4$-TEP/EC electrolyte.

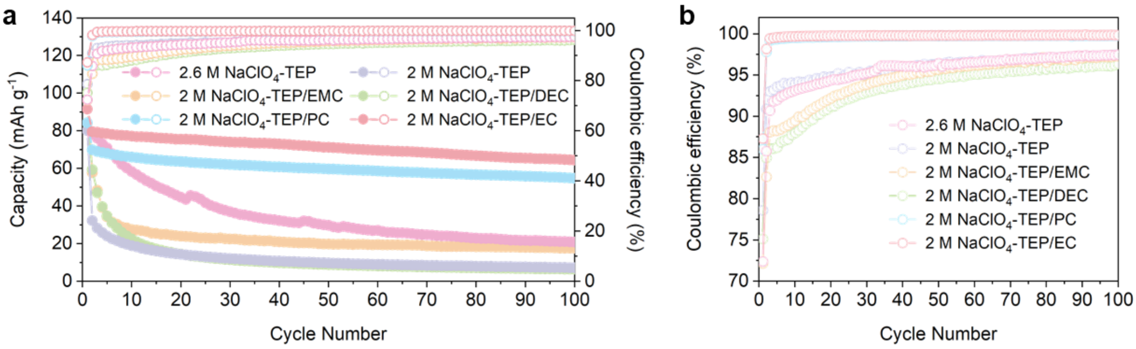

Supplementary Figure S33 (a) Cycling performance of RPB||HC coin cells using different electrolytes cycled at 50 mA g^{-1} after the first activation cycles at 20 mA g^{-1}, and (b) the corresponding amplified Coulombic efficiency.

Discussion: In comparison to the 2M $NaClO_4$-TEP electrolyte, it is evident that the RPB||HC cell in 2.6 M $NaClO_4$-TEP electrolyte with a higher concentration exhibits a decelerated degradation process. This can be attributed to the higher salt concentration, which effectively diminishes the number of free TEP molecules, consequently inhibiting their decomposition. However, due to the inadequate passivation capacity of ClO_4^-, it is difficult to maintain an effective robust electrode-electrolyte interphases to prevent TEP decomposition. This issue becomes even more pronounced in the electrode with higher mass loading, where the increased active reaction area intensifies the degradation process. In contrast, the 2M $NaClO_4$-TEP electrolyte containing cyclic carbonate additives exhibits stable cycling performance. This is due to the effective synergistic effect between cyclic carbonates and ClO_4^- in passivating the electrode, which effectively facilitates to form a stable passivation film and prevent further solvent decomposition.

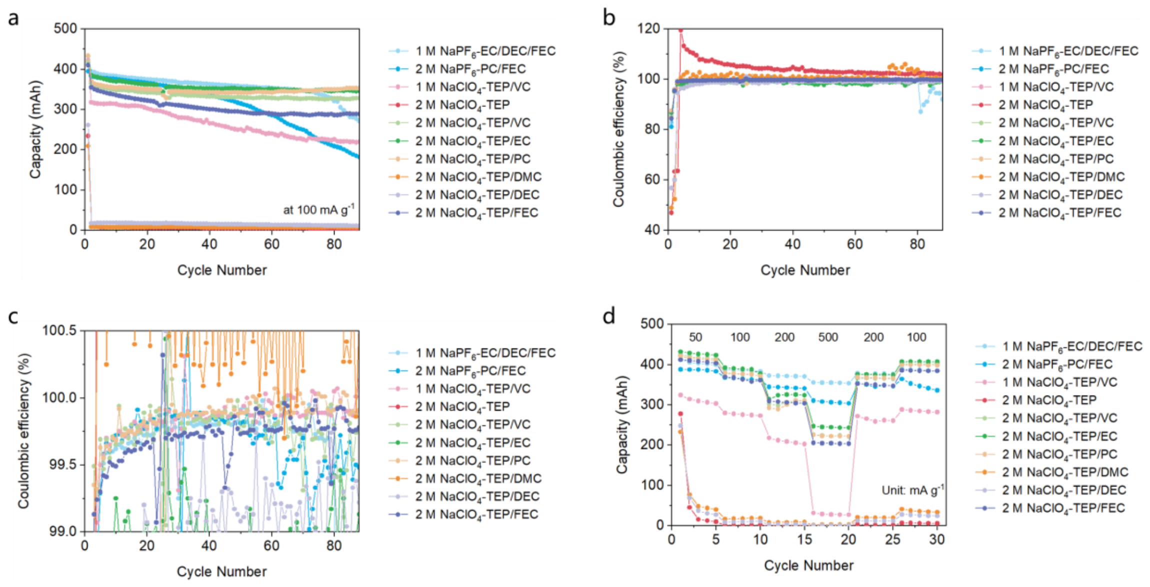

Supplementary Figure S34 Cycling performance (a), corresponding Coulombic efficiency (b, c) and rate performance (d) of RPB||HC pouch cells using 1 M $NaPF_6$-EC/DEC/FEC (EC:DEC=1:1 vol%, 5 wt% FEC), 2 M $NaPF_6$-PC/FEC (5 wt% FEC), 1 M $NaClO_4$-TEP/VC, 2 M $NaClO_4$-TEP, 2 M $NaClO_4$-TEP/VC, 2 M $NaClO_4$-TEP/EC, 2 M $NaClO_4$-TEP/PC, 2 M $NaClO_4$-TEP/DMC, 2 M $NaClO_4$-TEP/DEC, 2 M $NaClO_4$-TEP/FEC electrolyte.

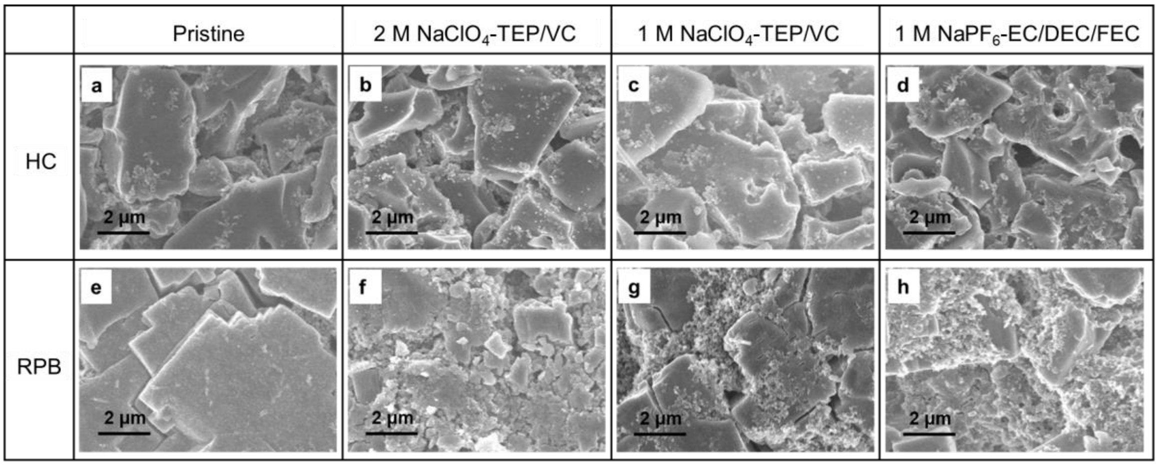

Supplementary Figure S35 SEM images of the RPB cathode and HC anode after 50 cycles in different electrolytes.

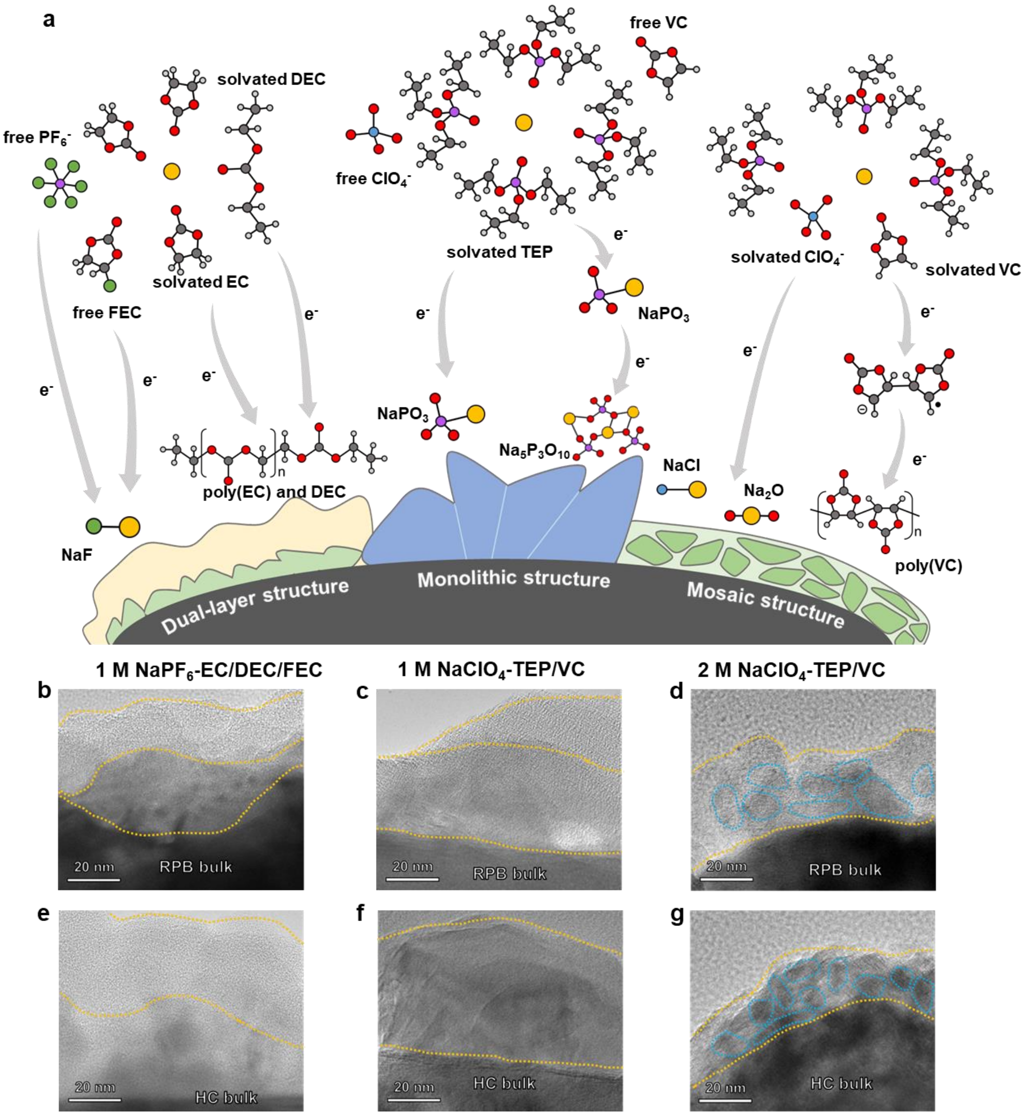

Supplementary Figure S36 Cryo-TEM of EEI formed on cathode and anode surface in various electrolytes. (a) Schematic illustration of the formation of EEI in various electrolyte. The (b) cathode and (e) anode surface in 1 M $NaPF_6$-EC/DEC/FEC electrolyte (dual-layer structure). The (c) cathode and (f) anode surface in 1 M $NaClO_4$-TEP/VC electrolyte (monolithic structure). The (d) cathode and (g) anode surface in 2 M $NaClO_4$-TEP/VC electrolyte (mosaic structure).

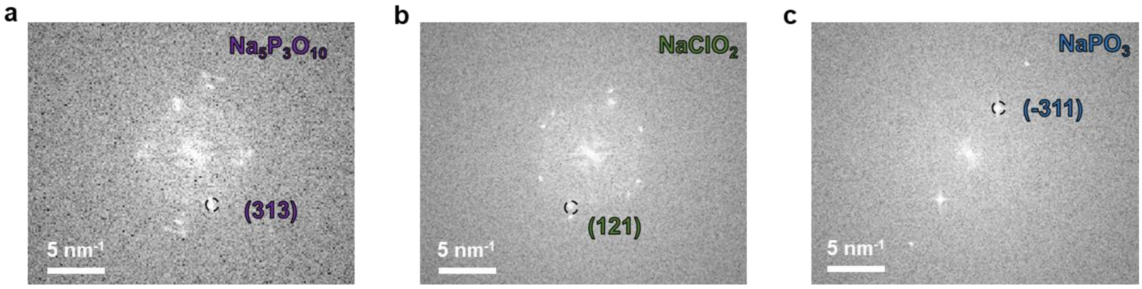

Supplementary Figure S37 Representative FFT of $Na_2P_3O_{10}$, $NaClO_2$, and $NaPO_3$ observed in the CEI and SEI of 2 M $NaClO_4$-TEP/VC electrolyte.

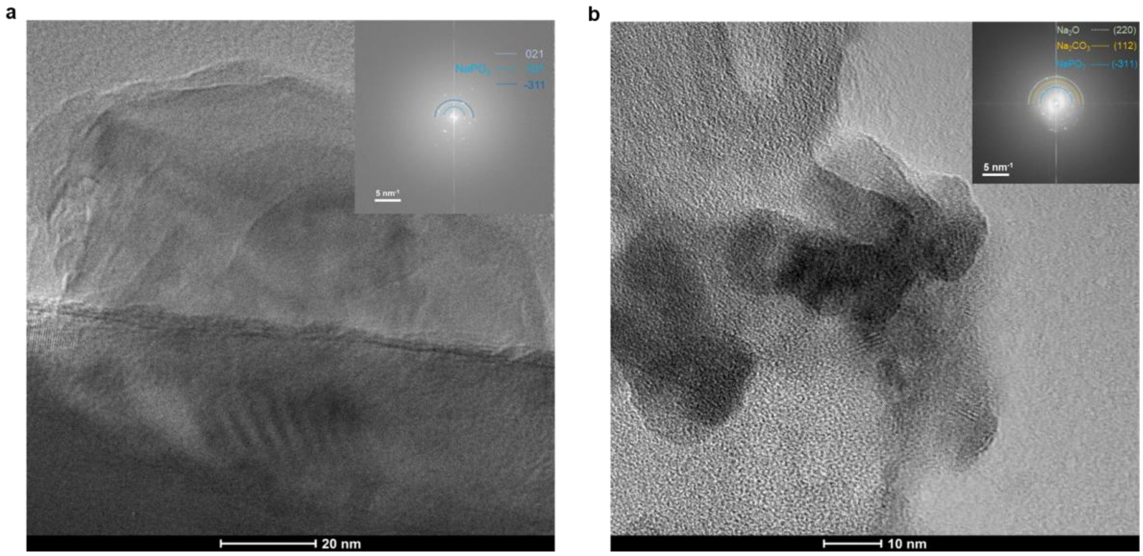

Supplementary Figure S38 Cryo-TEM and corresponding FFT of the (a) RPB cathode and (b) HC anode surface in 1 M $NaClO_4$-TEP/VC electrolyte.

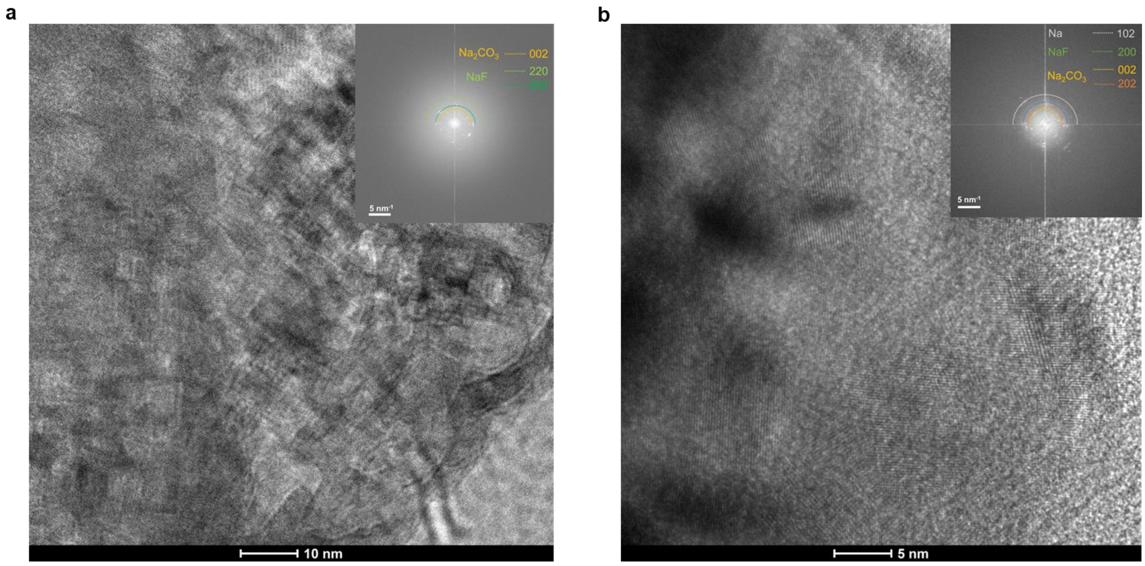

Supplementary Figure S39 Cryo-TEM and corresponding FFT of the (a) RPB cathode and (b) HC anode surface in 1 M $NaPF_6$-EC/DEC/FEC electrolyte.

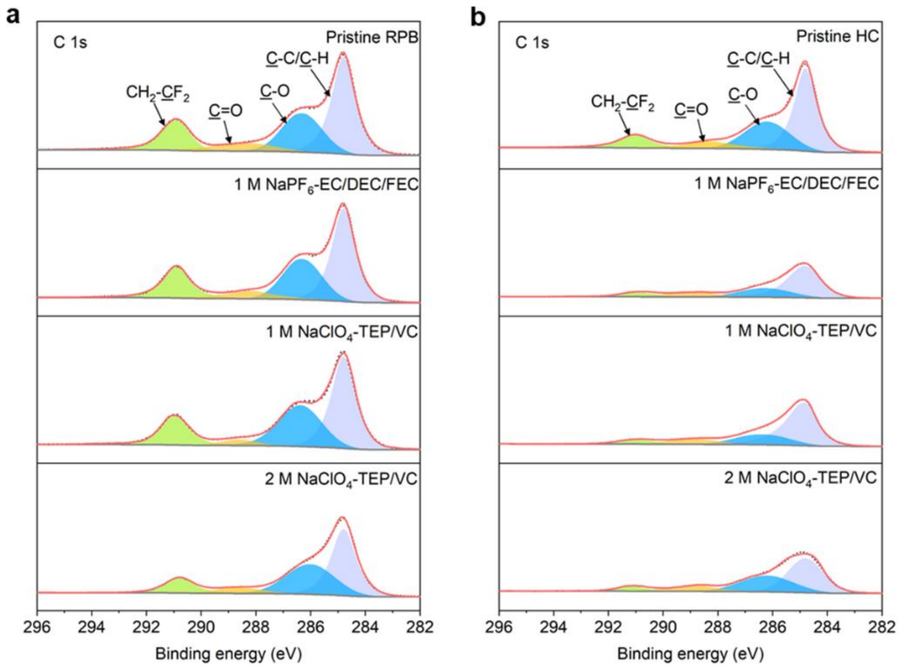

Supplementary Figure S40 XPS spectra of the cycled RPB cathodes (a) and cycled HC anodes (b) in 1 M $NaPF_6$-EC/DEC/FEC, 1 M $NaClO_4$-TEP/VC (1 M $NaClO_4$) and 2 M $NaClO_4$-TEP/VC (2 M $NaClO_4$) electrolyte.

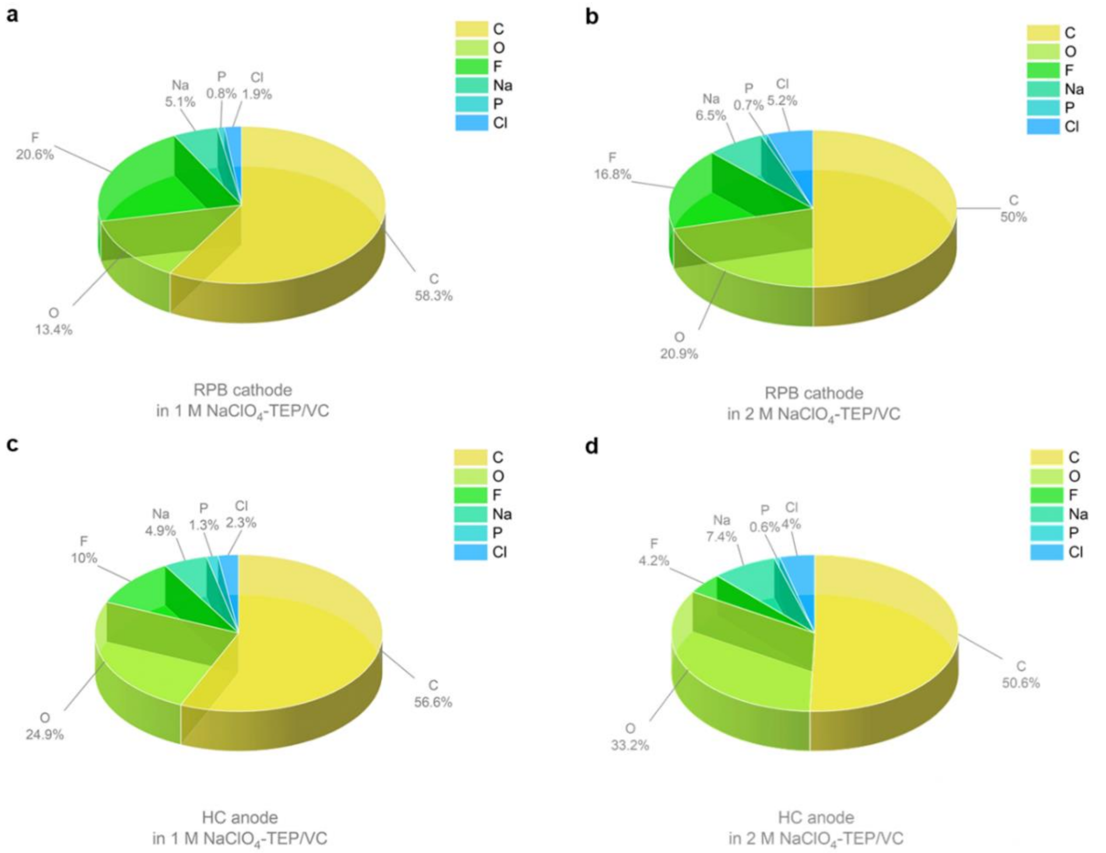

Supplementary Figure S41 Atomic concentrations from the C 1s, O 1s, F 1s, Na 1s, P 2p and Cl 2p spectra on the RPB cathode and HC anode in 1 M $NaClO_4$-TEP/VC and 2 M $NaClO_4$-TEP/VC electrolyte, respectively.

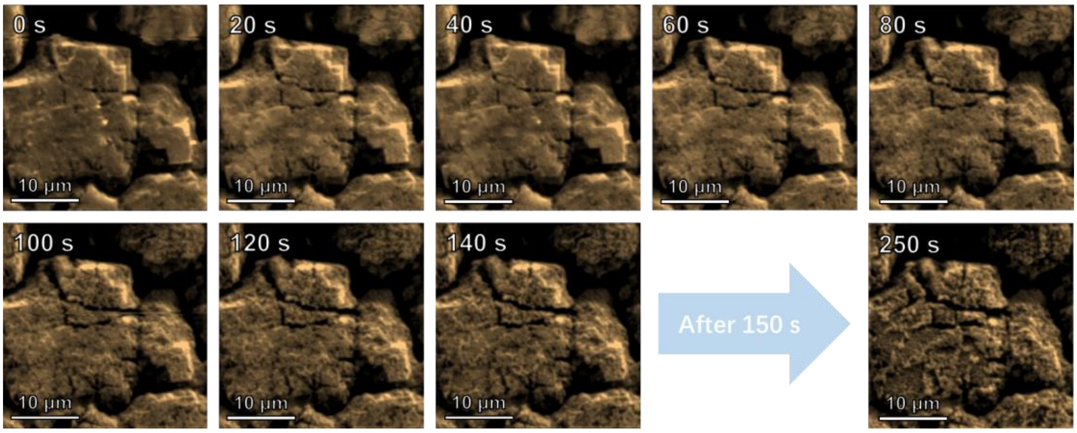

Supplementary Figure S42 Focused ion beam-scanning electron microscope (FIB-SEM) image for cathode.

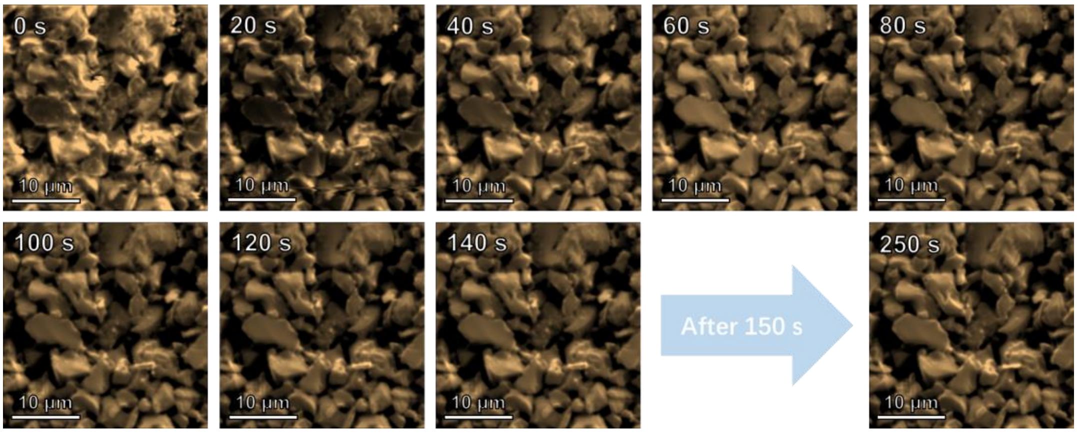

Supplementary Figure S43 Focused ion beam-scanning electron microscope (FIB-SEM) image for anode.

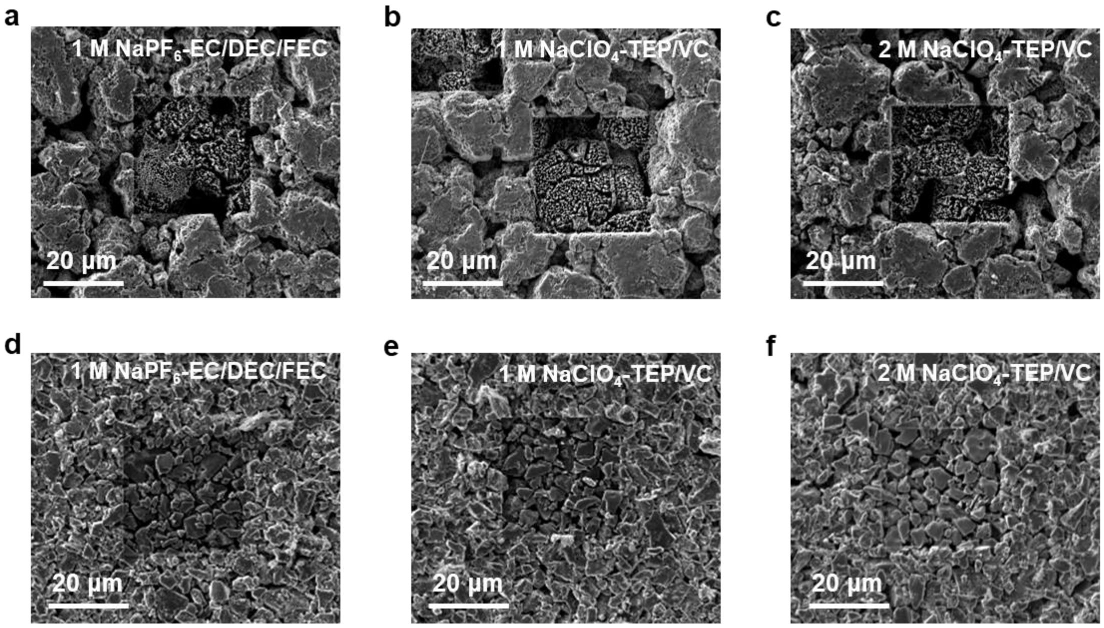

Supplementary Figure S44 SEM image for cathode (a-c) and anode (d-f) after FIB in various electrolytes.

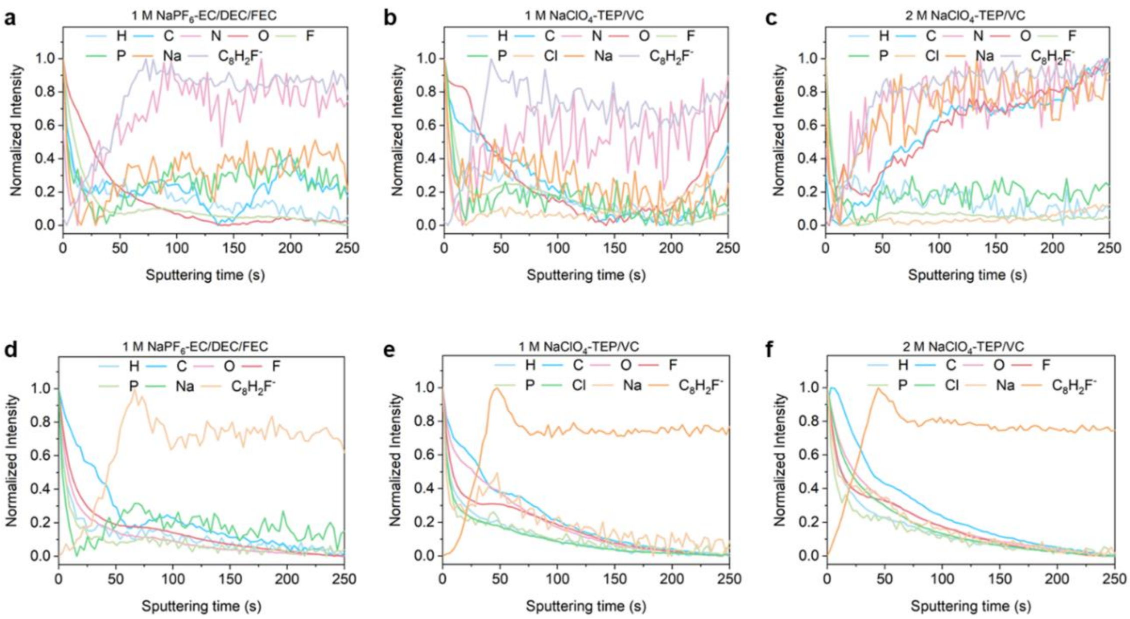

Supplementary Figure S45 Normalized depth profiling of all elements contained in (a-c) RPB cathode and (d-f) HC anode in various electrolytes (the $C_5H_2F^-$ from the binder).

Discussion: In the 2M $NaClO_4$-TEP/VC electrolyte, various ion fragment generated by sputtering at the RPB cathode and HC anodes shows a very similar trend with sputtering time, indicating the high uniformity of the formed EEI.

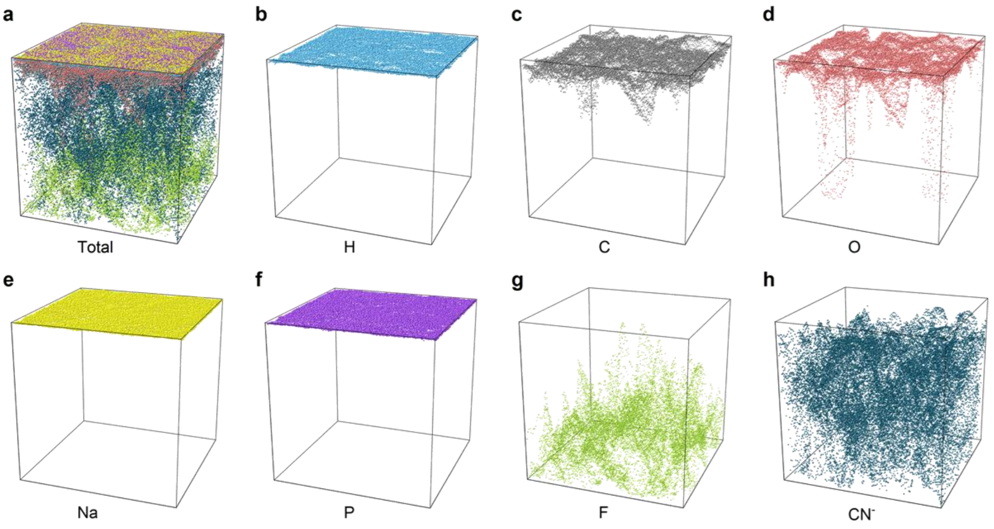

Supplementary Figure S46 3D spatial distribution of elements in CEI on the surface of RPB cathode using 1 M $NaPF_6$-EC/DEC/FEC electrolyte.

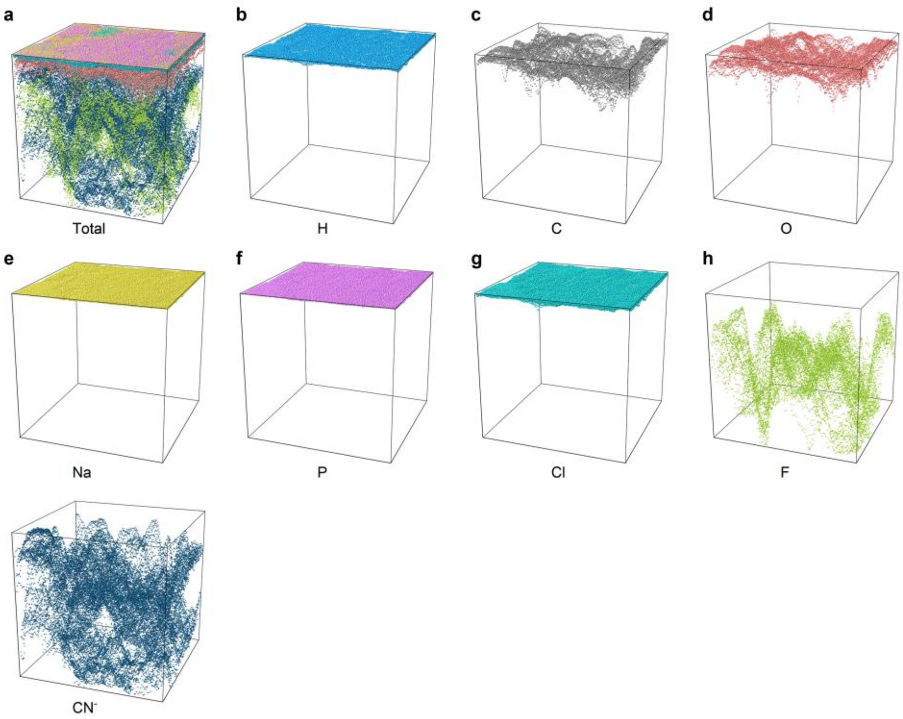

Supplementary Figure S47 3D spatial distribution of elements in CEI on the surface of RPB cathode using 1 M $NaClO_4$-TEP/VC electrolyte.

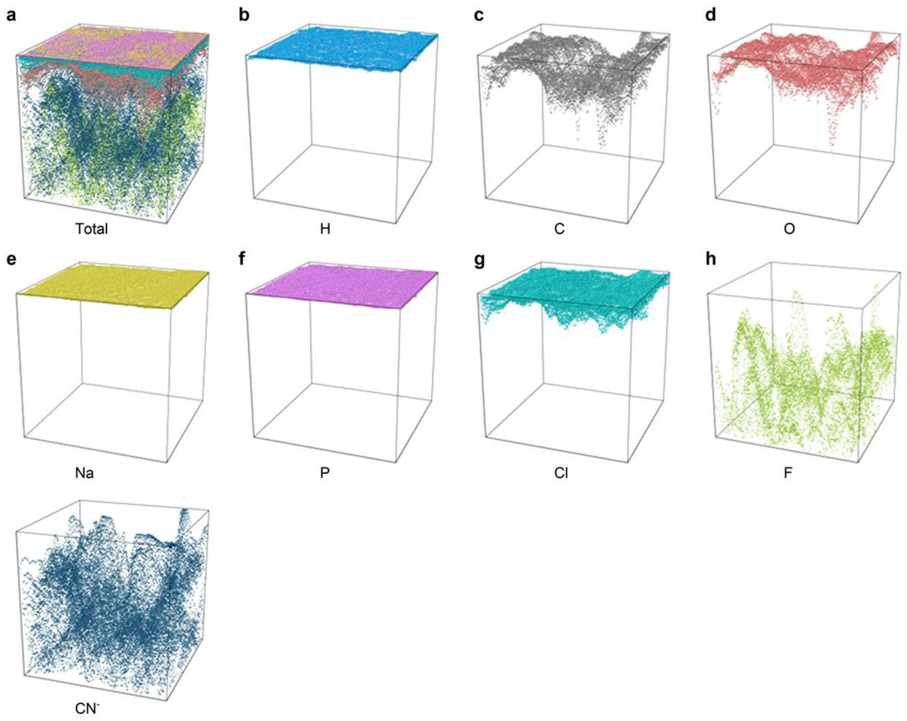

Supplementary Figure S48 3D spatial distribution of elements in CEI on the surface of RPB cathode using 2 M $NaClO_4$-TEP/VC electrolyte.

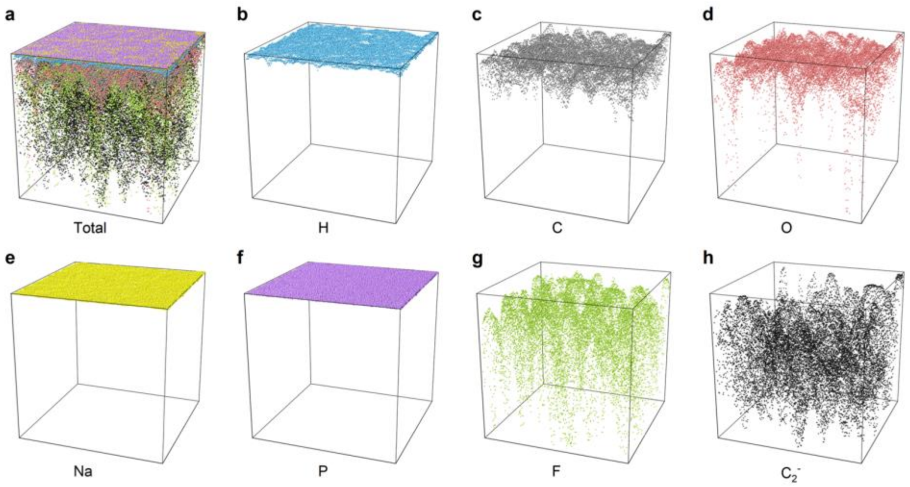

Supplementary Figure S49 3D spatial distribution of elements in SEI on the surface of HC anode using 1 M $NaPF_6$-EC/DEC/FEC electrolyte.

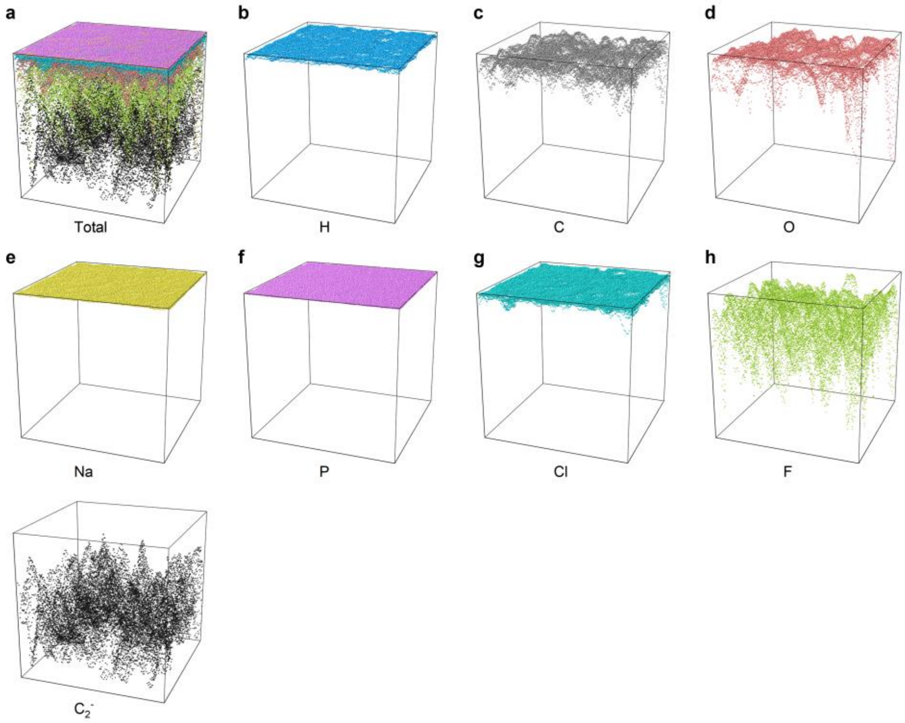

Supplementary Figure S50 3D spatial distribution of elements in SEI on the surface of HC anode using 1 M $NaClO_4$-TEP/VC electrolyte.

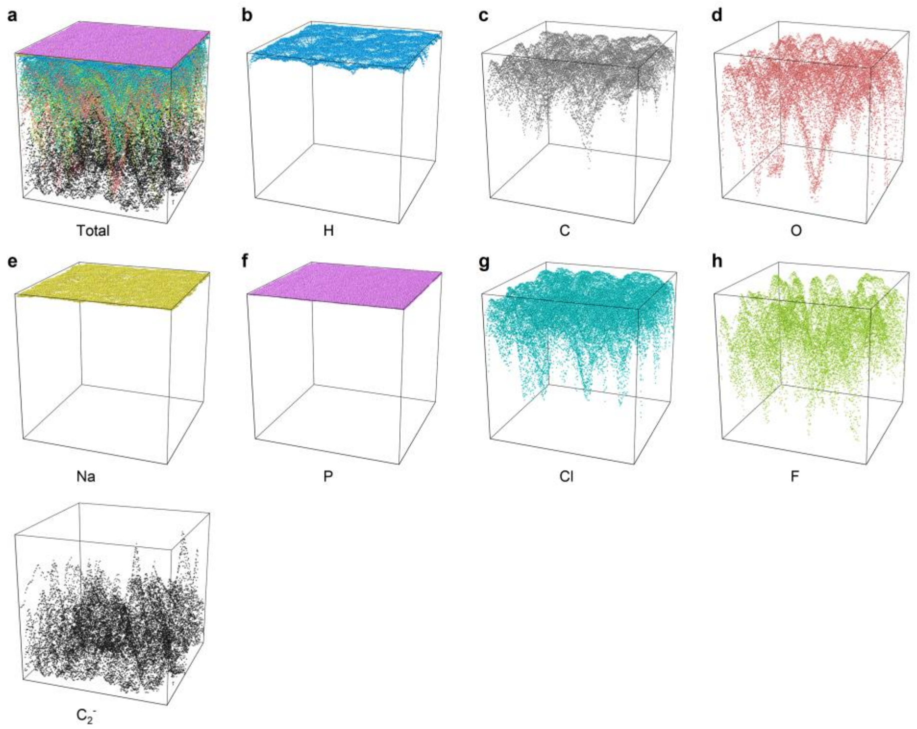

Supplementary Figure S51 3D spatial distribution of elements in SEI on the surface of HC anode using 2 M $NaClO_4$-TEP/VC electrolyte.

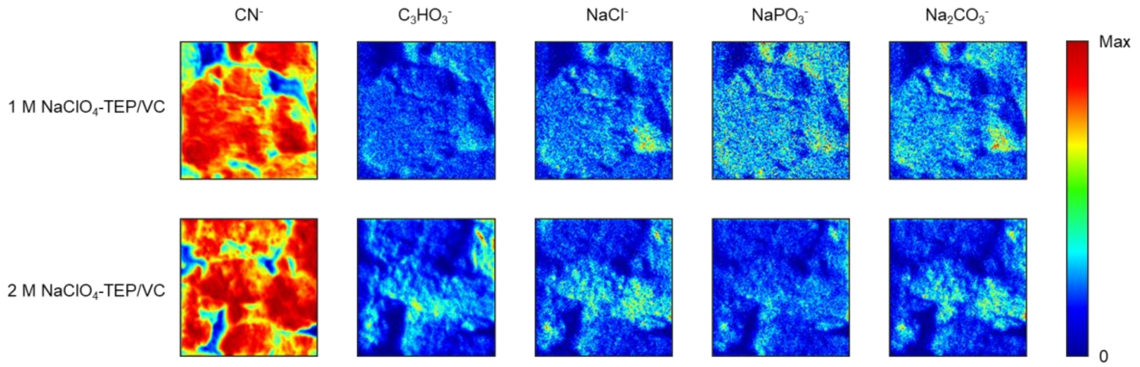

Supplementary Figure S52 The TOF-SIMS mapping distribution of related component in RPB cathode of 1 M $NaClO_4$-TEP/VC and 2 M $NaClO_4$-TEP/VC electrolyte, respectively.

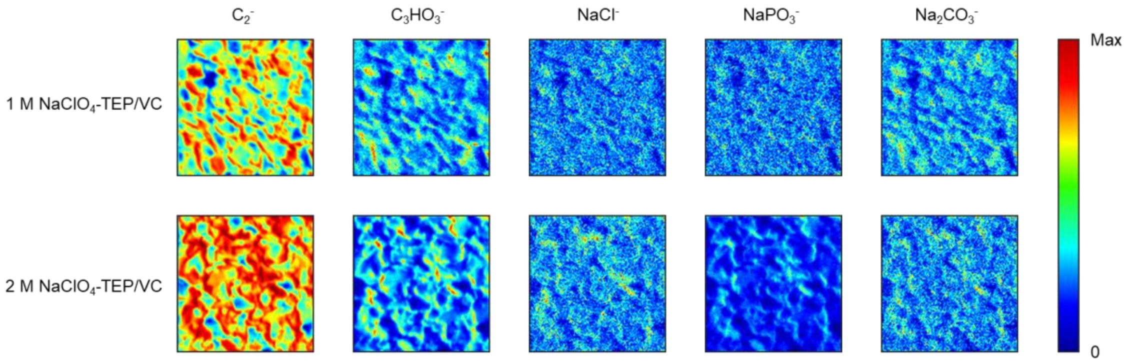

Supplementary Figure S53 The TOF-SIMS mapping distribution of related component in HC anode of 1 M $NaClO_4$-TEP/VC and 2 M $NaClO_4$-TEP/VC electrolyte, respectively.

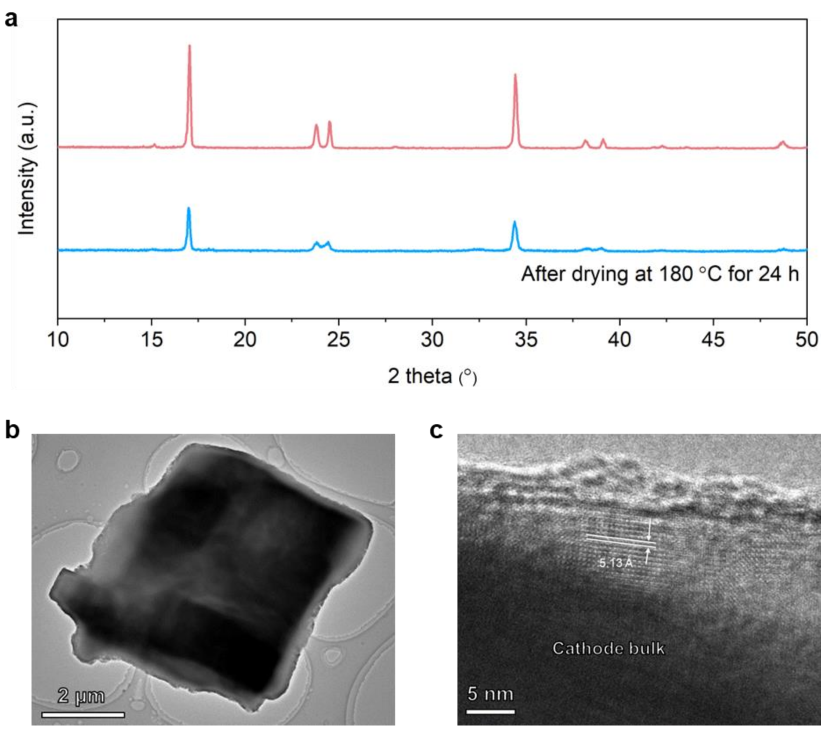

Supplementary Figure S54 Structural characterizations of the synthesized RPB (a) XRD pattern, (b, c) Cryo-TEM image.

Reference:

[1] W. Wang, Y. Gang, Z. Hu, Z. Yan, W. Li, Y. Li, Q. F. Gu, Z. Wang, S. L. Chou, H. K. Liu, S. X. Dou, *Nat. Commun.* **2020**, 11, 980.

[2] Yanai, T.; Tew, D. P.; Handy, N. C. A new hybrid exchange-correlation functional using the Coulomb-attenuating method (CAM-B3LYP). *Chem. Phys. Lett.* **2004**, 393, 51-57.

[3] Andersson, M. P.; Uvdal, P. New scale factors for harmonic vibrational frequencies using the B3LYP density functional method with the triple-ζ basis set 6-311+G(d,p). *J. Phys. Chem. A* **2005**, 109, 2937–2941.

[4] Duan, Y.; Wu, C.; Chowdhury, S.; Lee, M. C.; Xiong, G.; Zhang, W.; Yang, R.; Cieplak, P.; Luo, R.; Lee, T.; Caldwell, J.; Wang, J.; Kollman, P. A point-charge force field for molecular mechanics simulations of proteins based on condensed-phase quantum mechanical calculations. *J. Comput. Chem.* **2003**, 24 (16), 1999–2012.

[5] Berendsen, H.J.C.; van der Spoel, D.; van Drunen, R. GROMACS: A message-passing parallel molecular dynamics implementation. *J. Comput. Chem.* **1995**, 91, 43–56.

[6] Sousa da Silva, Alan W.; Vranken, W. F. ACPYPE-antechamber python parser interface. *BMC Res. Notes* **2012**, 5, 367.
